# Electrocautery smoke exposure and efficacy of smoke evacuation systems in minimally invasive and open surgery: a prospective randomized study

**DOI:** 10.1038/s41598-022-08970-y

**Published:** 2022-03-23

**Authors:** Gregor J. Kocher, Abigail R. Koss, Michael Groessl, Joerg C. Schefold, Markus M. Luedi, Christopher Quapp, Patrick Dorn, Jon Lutz, Luca Cappellin, Manuel Hutterli, Felipe D. Lopez-Hilfiker, Mohammad Al-Hurani, Sergio B. Sesia

**Affiliations:** 1 Division of General Thoracic Surgery, Inselspital, Bern University Hospital, University of Bern, 3010 Bern, Switzerland; 2 Tofwerk AG, Thun, Switzerland; 3 Department of Nephrology and Hypertension and Department of BioMedical Research, Inselspital, Bern University Hospital, University of Bern, Bern, Switzerland; 4 Department of Intensive Care Medicine, Inselspital, Bern University Hospital, University of Bern, Bern, Switzerland; 5 Department of Anesthesiology and Pain Medicine, Inselspital, Bern University Hospital, University of Bern, Bern, Switzerland; 6 Department of General and Special Surgery, Faculty of Medicine, The Hashemite University, Zarqa, Jordan

**Keywords:** Cancer, Diseases, Health care, Health occupations, Medical research, Risk factors

## Abstract

Worldwide, health care professionals working in operating rooms (ORs) are exposed to electrocautery smoke on a daily basis. Aims of this study were to determine composition and concentrations of electrocautery smoke in the OR using mass spectrometry. Prospective observational study at a tertiary care academic center, involving 122 surgical procedures of which 84 were 1:1 computer randomized to smoke evacuation system (SES) versus no SES use. Irritating, toxic, carcinogenic and mutagenic VOCs were observed in OR air, with some exceeding permissible exposure limits (OSHA/NIOSH). Mean total concentration of harmful compounds was 272.69 ppb (± 189 ppb) with a maximum total concentration of harmful substances of 8991 ppb (at surgeon level, no SES). Maximum total VOC concentrations were 1.6 ± 1.2 ppm (minimally-invasive surgery) and 2.1 ± 1.5 ppm (open surgery), and total maximum VOC concentrations were 1.8 ± 1.3 ppm at the OR table ‘at surgeon level’ and 1.4 ± 1.0 ppm ‘in OR room air’ away from the operating table. Neither difference was statistically significant. In open surgery, SES significantly reduced maximum concentrations of specific VOCs at surgeon level, including aromatics and aldehydes. Our data indicate relevant exposure of health care professionals to volatile organic compounds in the OR. Surgical technique and distance to cautery devices did not significantly reduce exposure. SES reduced exposure to specific harmful VOC’s during open surgery.

Trial Registration Number: NCT03924206 (clinicaltrials.gov).

## Introduction

Electrocautery devices are among the most efficient tools for dividing tissue and providing hemostasis, and are routinely used in most surgical interventions. Nevertheless, exposure to electrocautery smoke is associated with potential consequences that affect hundreds of thousands of healthcare workers each year. These include asthma, emphysema, chronic bronchitis, hypoxia and dizziness, nose and throat irritation, eye irritation, carcinoma of the respiratory tract, leukemia, cardiovascular dysfunction, headache, hepatitis, allergies, and others^1–3^.

Tomita et al. showed that fumes released into ambient air during surgery when using electrosurgical instruments may be as mutagenic as cigarette smoke^4^. Due to carcinogens such as 1,2-dichloroethane, benzene, and polycyclic aromatic hydrocarbons^5^, the average daily impact of electrocautery smoke on a given OR team was estimated to be equivalent to 27–30 unfiltered cigarettes^2^. Electrocautery smoke has also been shown to carry infectious material such as viruses (including SARS-CoV-2 and human papilloma virus), bacteria, and/or viable cells^5–7^.

For this reason, many occupational safety and health institutes advise the use of smoke evacuation systems (SES), but adherence to these recommendations is reported to be low (14% with electrocautery use and 47% during open laser surgery^8^).

Reluctance to use SES might be attributable to the fact that only sparse real-life data on the effectiveness of these devices is available. In a recent wet lab study, we showed that proton-transfer-reaction (PTR) mass spectrometry allows for real-time measurement of electrocautery smoke composition. We observed that routine surgical masks do not protect against this type of smoke because hazardous particles are too small, and that SES might be able to reduce hazardous compounds at least to a certain degree. We also detected significant concentrations of hazardous substances at the exhaust of the SES, despite having passed a pre-filter, an Ultra-Low Particulate Air filter (ULPA), and an active carbon filter^9^.

Based on the data that has been published so far, including our own, we therefore undertook an investigation of the composition and concentration of electrocautery smoke in an operating theater under real-life conditions.

The main aims of our study were to measure the concentrations of harmful volatile organic compounds (VOC’s) in the OR and evaluate the effectiveness of SES in daily practice as well as to investigate whether the surgical approach (i.e., open vs. minimally invasive) and the distance to the cautery source have an impact on the exposure of OR personnel to surgical smoke.

## Methods

The primary endpoint of our study was to determine the concentration of hazardous VOC’s in the OR at surgeon level during surgery with electrocautery devices. Operations were 1:1 randomized into SES use vs. no SES use and prospectively recorded in order to be able to compare measurements with and without the use of SES. Secondary endpoints included ‘direct’ (operating team at OR table – labelled ‘at surgeon level) vs. ‘indirect’ (OR personnel distant from the operating table – labelled ‘in room air’) smoke exposure, and comparison of smoke exposure depending on the operative technique used (open vs. minimally invasive surgery). The study was performed during a four-month period (May 2019 to September 2019) in a dedicated OR of a tertiary care academic center (Division of General Thoracic Surgery at the Inselspital, Bern University Hospital, University of Bern, Switzerland). Consecutive surgical procedures with a planned duration of > 1 h were included. Exclusion criteria were a contraindication for electrocautery use (i.e., patients with an implanted cardioverter-defibrillator (ICD) and/or an implanted neurostimulator).

Formal ethical approval was waived by the cantonal ethics committee (Kantonale Ethikkommission, KEK, Bern) since no patient data was used for this study and only the type of procedure (open or minimally invasive surgery) was recorded. The study was registered on clinicaltrials.gov (NCT03924206). All medical professionals participating provided written informed consent. The study was conducted in adherence to the Declaration of Helsinki.

### Types of operations

Surgical interventions were performed by the same surgical team in an OR meeting the latest standard requirements, including a vertical laminar flow (0.3–0.6 m/s) and anesthetic gas scavenging system. Mainly anatomical lung resections were performed. The decision to perform the procedure ‘open’ or ‘minimally invasive’ was made by the independent operating surgeon before randomization to surgery with or without SES. ‘Open surgery’ included an access to the operative field via a muscle-sparing anterolateral thoracotomy. ‘Minimally invasive surgery’ comprised operations done by VATS (video-assisted thoracic surgery), using a uniportal VATS technique with a 3–4 cm incision in the midaxillary line in the 5th intercostal space and use of a soft wound protector (no rib spreader, no CO2-Insufflation).

### Randomization and sample size

**Figure 1:**
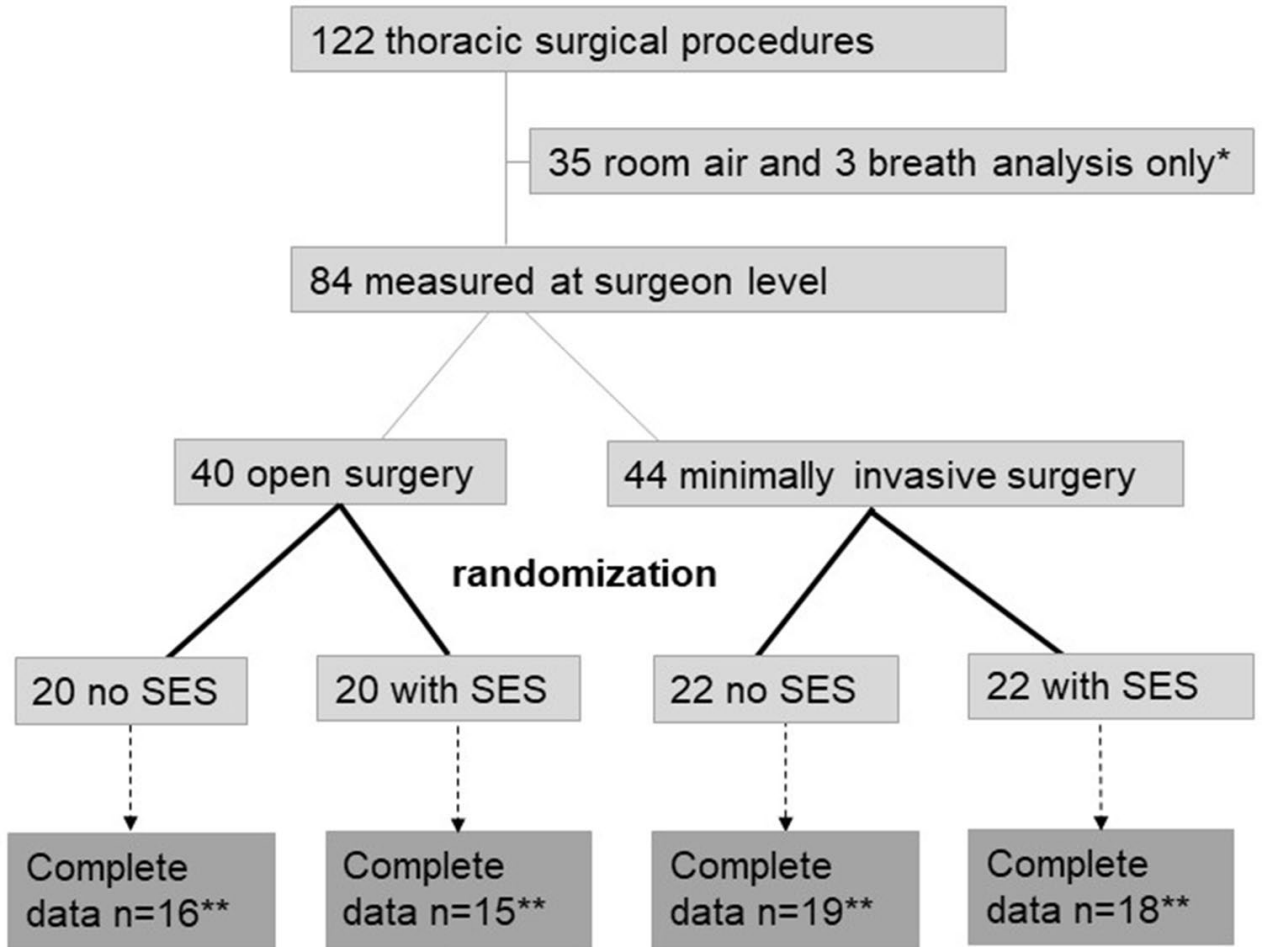
Flow chart overview of data analysis. *1 breath analysis was excluded for technical reasons. **In total, 17 datasets had to be excluded because of incomplete data (n = 6) or for technical reasons (n = 11).

Open and minimally invasive procedures were randomized (1:1, computer-based randomization in blocks of 4) to surgery with or without SES after the type of surgical procedure (open vs. minimally invasive) was determined (Fig. 1). The power analysis was based on previous data^10^: Concentrations of butadiene, benzene and furfural with and without SES were used when cutting muscle tissue and corrected for distance (2 × distance, assuming ½ of concentrations). Alpha was set to 0.05 and study power to 90%, indicating that a sample size of 15 surgeries was required in the ‘open surgery’ group.

### Additional measurements

During a total of 35 surgical procedures (open and minimally invasive), the OR room air away from the operating table was measured by placing the inlet of the MS right on top of the MS itself (approx. 150 cm above the ground and at least with 200 cm distance from the OR table).

In addition, during a total of 3 open procedures, the air that was inhaled and exhaled by the operating surgeon was measured by means of a dedicated face mask (see further details below and in Fig. 3).

### Electrocautery smoke evacuation system (SES)

**Figure 2:**
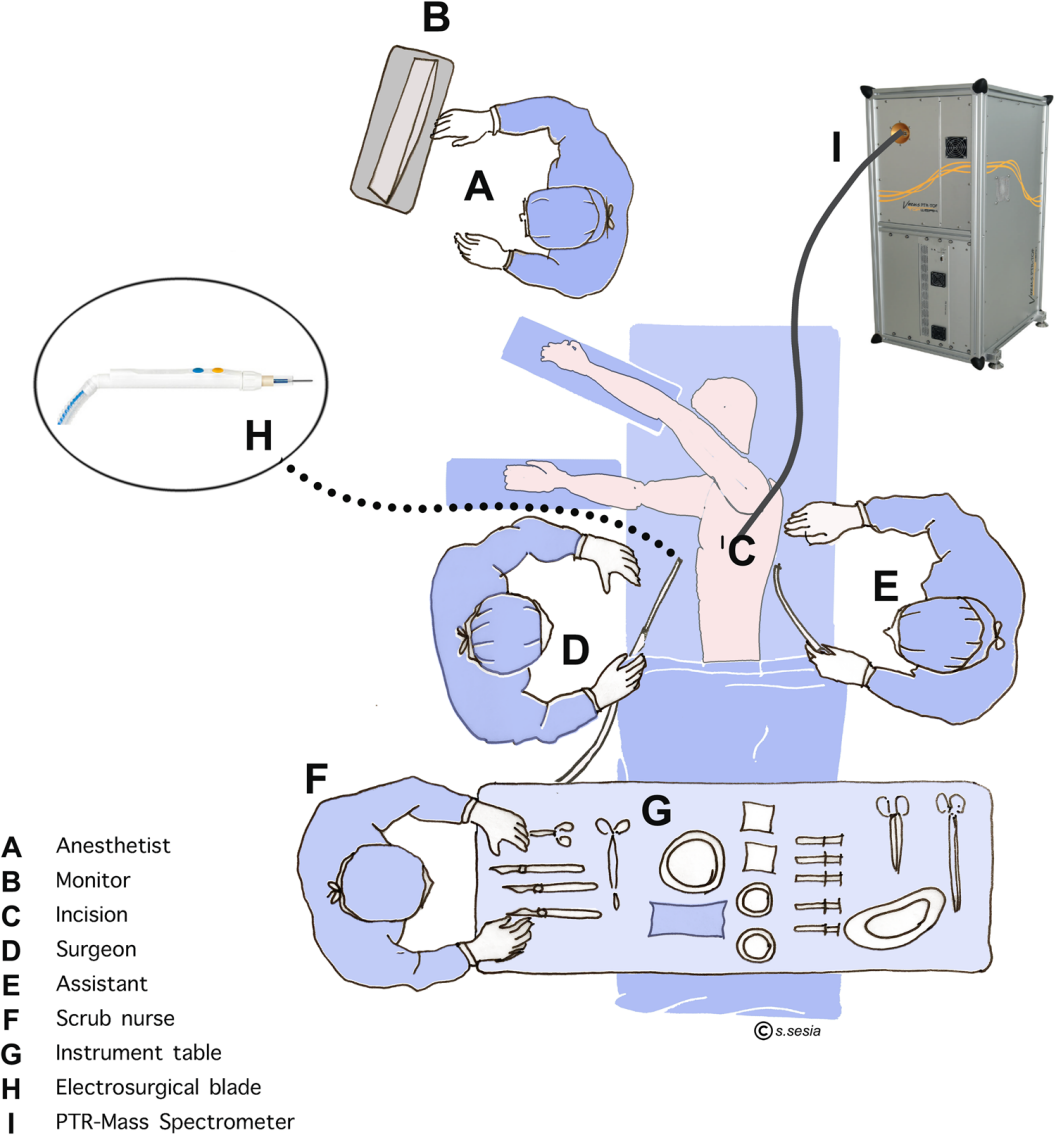
OR set-up for measurements at surgeon level. The inlet of the mass spectrometer (I) was placed at the level of the surgeon’s (D) mouth/nose, directly above the operative field (C). (Figure created with Adobe Photoshop version 22.4.2; https://www.adobe.com/products/photoshop.html).

A standard monopolar cautery device (VIO 300D, ERBE Swiss AG, Winterthur, Switzerland) was used with the following settings: *coagulation*: spray, effect 2, 80 W; and *cut*: effect 4, 100 W. In the group without SES, a standard cautery pencil was used and the released smoke was cleared as usual, using a fluid suction with a Yankauer plastic tip. In the group ‘with SES’, a mobile SES (IES 2, ERBE Swiss AG, Winterthur, Switzerland) was applied, consisting of a dedicated pencil with an integrated aspirator near the electrocautery tip. From the cautery pencil, there is a standard electrical plug and an additional aspirator connector leading to the smoke evacuator machine (Fig. 2). The smoke evacuator is automatically activated as soon as the cautery is used. The IES 2 has a suction output of > 550 l/min and was run at 100% suction output during our study, resulting in a flow of 110 l/min at the inlet on the cautery device. The evacuated air runs through a three-stage filtration process: first a ‘pre-filter’ removes large particles and liquid components, then a ULPA (Ultra-Low Penetration Air) filter captures fine particles and micro-organisms of up to 0.1 μm with a 99.9995% efficiency rate, and finally a high-performance activated carbon filter part is supposed to absorb odors and VOCs.

### Mass spectrometry measurements

Gas-phase VOC’s were measured using real-time proton-transfer-reaction mass spectrometry (Vocus S PTR-TOF, Tofwerk AG, Switzerland). VOCs were ionized via low-energy chemical ionization and identified based on exact mass. The instrument operated at a 1 Hz measurement frequency, measuring a mass range of 0–800 Th, with a resolving power of 5000 (FWHM m/dm). Before each operation, a 1-min instrument blank, 1-min calibration, and second 1-min instrument blank were performed. Similarly, after each operation, a 90-s instrument blank was performed, followed by a 10 min calibration using a standard gas mixture. Instrument background was subtracted using the pre-operation measurements. The mass spectrometer (MS) sampled through a 2.5 m 1/8″ inner diameter PFA inlet, heated to 80 °C in order to avoid condensation of VOCs at the level of the inlet and tubing before reaching the measurement chamber of the MS. The tip of the inlet was placed either above the surgical incision at the level of the surgeon’s mouth and nose (“at surgeon level”) or on top of the instrument, away from the operating table at the back of the OR (“room air”).

### Inspiratory/expiratory breath analysis

**Figure 3:**
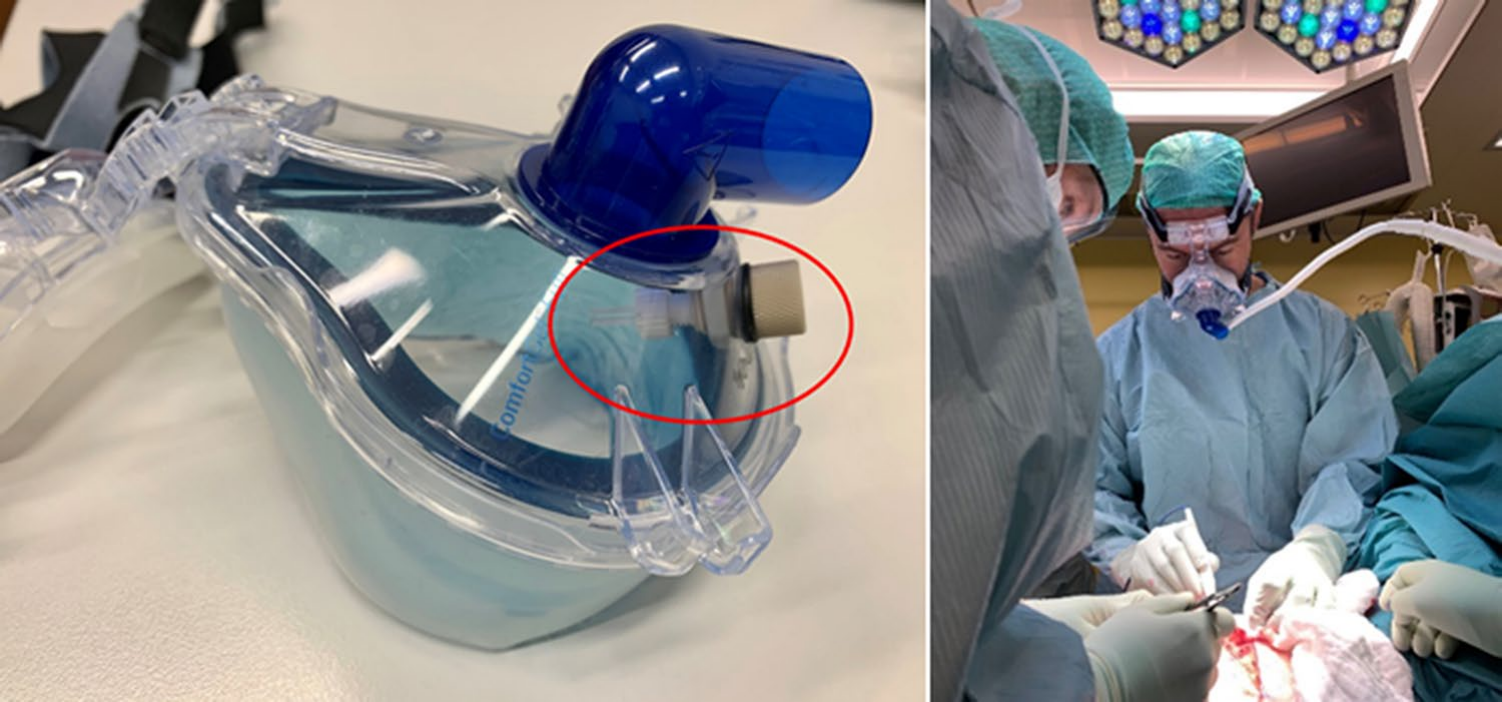
Face mask with dedicated connector for the mass spectrometry inlet (red circle) allowed measurement of inhaled and exhaled VOC’s during surgery.

To analyze the inspiratory and expiratory breath of surgeons, a dedicated full face mask (Full Face Mask Comfort Gel Blue, Philips Respironics, Philips AG, Horgen, Switzerland) with inlet/outlet MS measurements was initially used. The heated inlet was attached directly to the face mask (Fig. 3). Only three operations were monitored using the face mask because it obstructs the view of the surgeon and hampers the ability to perform or assist surgery. Exposure of the surgeon to a particular compound is related to the compound concentrations in air, minute ventilation, and time of exposure. The amount of a VOC inhaled was calculated at an assumed minute ventilation of 5 L and an average operation time of 120 min.

### Statistical analysis

The use of SES during the two different types of surgeries (open and minimally invasive) was randomized in order to decrease the effect of confounding variables.

Univariate analysis of the different settings was performed to determine mean total VOC concentrations as well as average maximum total VOC concentrations of the different settings (open surgery at surgeon level with SES and without SES, minimally invasive surgery at surgeon level with and without SES, room air measurements etc.).

We then used multiple linear regression analysis to separate effects of each experimental parameter. Each experimental parameter (with SES, without SES, in room air, at surgeon level, open, and minimally invasive) was assigned a value of zero or one for each operation; then, a least-squares fit was performed to quantify the effect on VOC concentrations related to each parameter. The linear regression analysis indicates that these experimental parameters only explain a small portion of the variability in the measured maximum and mean total VOC (R = 0.17). However, the experimental parameters can explain a larger portion of the variability in the mean concentrations of many individual VOCs (on average, R = 0.3).

## Results

Electrocautery smoke concentrations were measured during a total of 122 surgical procedures. Data from 105 operations remained in the final data set (Fig. 1), with n = 17 excluded for technical reasons. Of those, n = 6 had incomplete data and n = 11 had performance-critical instrument parameters out of tolerance.

### Mass spectrometry

Approximately 1300 ion masses were quantified using Tofware data processing software (Tofwerk, Switzerland). After excluding primary ions, known common fragments, and ions with very small signal or low estimated rate constants, 1064 ion masses (representing individual VOCs) were selected for analysis. Total VOC concentrations, defined as the sum of the 1064 VOCs quantified by PTR-MS, had a typical average concentration of 403 parts-per-billion by volume (ppb), and a typical maximum concentration of 1.7 parts-per-million (supplementary Fig. 1). We also considered the total concentration of VOCs that are known to be carcinogenic, mutagenic, irritating, and/or toxic (Table 1). The latter had an average concentration (at surgeon level, no SES) of 272.69 ppb, and a maximum concentration of 8991 ppb. Although only a few substances (including Formaldehyde, Methanol and 1,3-butadiene/1-Butyne) exceeded the exposure limits as defined by the NIOSH/OSHA^14^, the measured cumulative maximum concentrations of known harmful VOC’s reached up to almost 9 ppm for open surgeries when no SES was used. The statistics include all measured operations except for breath analysis. The VOC mixture was dominated by small oxygenated VOCs, especially formaldehyde, methanol, acetaldehyde, acetone, acrolein, ethanol, and 2-butadione (supplementary Fig. 2).

**Table 1:** Harmful volatile compounds measured without SES at surgeon level (respective concentrations are given).

VOC name	CAS number	Potential health effects	Average detected concentration (ppb)	Maximum detected concentration (ppb)	Maximum permissible exposure limit (ppb)^14^
Hydrogen cyanide	74-90-8	Acute toxic	0.58	22.71	4700
Formaldehyde	50-00-0	Carcinogenic, mutagenic, acute toxic, corrosive	9.12	1721.95	750 (OSHA) 16 (NIOSH)
Methanol	67-56-1	Acute toxic	5.06	287.34	200
Acetonitrile	75-05-8	Acute toxic, irritant, odor	1.11	55.38	40,000 (OSHA) 20,000 (NIOSH)
Acetaldehyde	75-07-0	Carcinogenic, mutagenic, irritant, odor	141.61	739.66	25,000
Formic acid	64-18-6	Acute toxic, corrosive, odor	0.15	19.35	5,000
Ethanol	64-17-5	Irritant, odor	34.84	2054.08	100,0000
Methanethiol	74-93-1	Acute toxic, odor	0.01	7.56	500
Acrylonitrile	107-13-1	Carcinogenic, acute toxic, irritant, corrosive, odor	0.02	14.57	2,000 (OSHA) 1,000 (NIOSH)
1,3-butadiene/1-Butyne	106-99-0 107-00-6	Carcinogenic, mutagenic, odor	16.89	1066.49	1,000
Propionitrile	107-12-0	Acute toxic, irritant, odor	0.03	13.63	6,000
Butenes	106-98-9 624-64-6 590-18-1 115-11-7	Irritant, odor	5.22	128.66	
Acetone/Propanal	67-64-1 123-38-6	Irritant, odor	42.17	521.66	1,000,000
3-butenenitrile/pyrrole	109-75-1 109-97-7	Acute toxic, odor, mutagen	0.02	20.49	
1,3-pentadiene	2004-70-8	Odor, possible carcinogen	3.25	196.38	
Crotonaldehyde	123-73-9	Acute toxic, irritant, corrosive, odor	0.39	12.05	2,000
Benzene	71-43-2	Carcinogen, mutagen, irritant, odor	0.31	35.14	1,000 (OSHA) 100 (NIOSH)
Pyridine	110-86-1	Acute toxic, irritant, odor	0.02	2.61	5,000
2-Methylbutanenitrile (2-Methylbutyronitrile) /Isovaleronitrile (3-Methylbutyrolnitrile)	18936-17-9 / 625-28-5	Acute toxic, irritant, odor	0.02	5.30	
3-Methyl-2-butenal	107-86-8	Acute toxic, irritant, corrosive, odor	0.11	2.96	
gamma-Butyrolactone	96-48-0	Acute toxic, irritant, corrosive	0.62	4.34	50,000
Isovaleraldehyde (3-Methylbutanal) / 2-Methylbutyraldehyde (2-Methylbutanal) / Pentanal (Valeraldehyde)	590-86-3 96-17-3 110-62-3	Acute toxic, irritant, odor	0.62	128.95	
Toluene	108-88-3	Teratogenic, irritant, odor	0.49	123.60	200,000
Phenol	108-95-2	Mutagen, acute toxic, corrosive, odor	2.84	10.44	5,000
Furfural	98-01-1	Carcinogenic, acute toxic, odor	0.07	3.07	2,000
2,5-dimethylfuran	625-86-5	Irritant, odor	0.07	1.74	
Methyl methacrylate	80-62-6	Irritant, odor	0.33	2.13	100,000
Styrene	100-42-5	Teratogenic, carcinogenic, irritant, odor	0.13	2.51	100,000
2,3-Dihydro indene (Indane) / 3-Methylstyrene (1-Ethenyl-3-methylbenzene)	496-11-7 100-80-1	Irritant, odor	0.09	1.48	
n-Propylbenzene (Pseudocumene)	103-65-1	Irritant, odor	0.15	24.97	
Desflurane	57041-67-5	Irritant, odor	1.46	1002.03	
Sevoflurane	28523-86-6	Irritant, odor	4.91	757.78	
*Total of irritating, toxic, carcinogenic and/or mutagenic compounds*			272.69	8991	

### Volatile organic compounds (VOC) of specific interest

VOCs with known or suspected harmful health effects were detected. A list of compounds of interest was compiled by considering VOCs previously indicated in surgical smoke according to the literature: VOCs which may have harmful health effects according to the European CMR Rating, the WHO International Agency for Research on Cancer, or the GHS hazard statement, and VOCs which have a strong odor. An overview of potentially harmful compounds found at significant concentrations in smoke is given in Table 1. Reported concentrations are average and maximum levels across all operations. Concentrations of additional detected VOC are reported in supplementary Table 1.

### Effects of operation type, measurement location, and smoke evacuation system

Concentrations of individual harmful VOCs and total VOC were assessed. We investigated effects on maximum and mean concentrations. Table 1 comprises average as well as maximum detected concentrations of all measured harmful VOC’s during all thoracic surgical procedures at surgeon level. Since our main aim was to detect VOC concentrations at surgeon level and evaluate the effectiveness of the smoke evacuation system, we first took a look at these results. In respect to the pre-study power analysis, the minimum number of measurements was met with 16 (no SES) and 15 (with SES) complete data sets in the open group.

When comparing average VOC concentrations at surgeon level in all surgeries without SES (272.69 ± 188.9), to the situation at surgeon level with SES (247.22 ± 145.11), the difference was not significant (*p* = 0.53).

**Figure 4:**
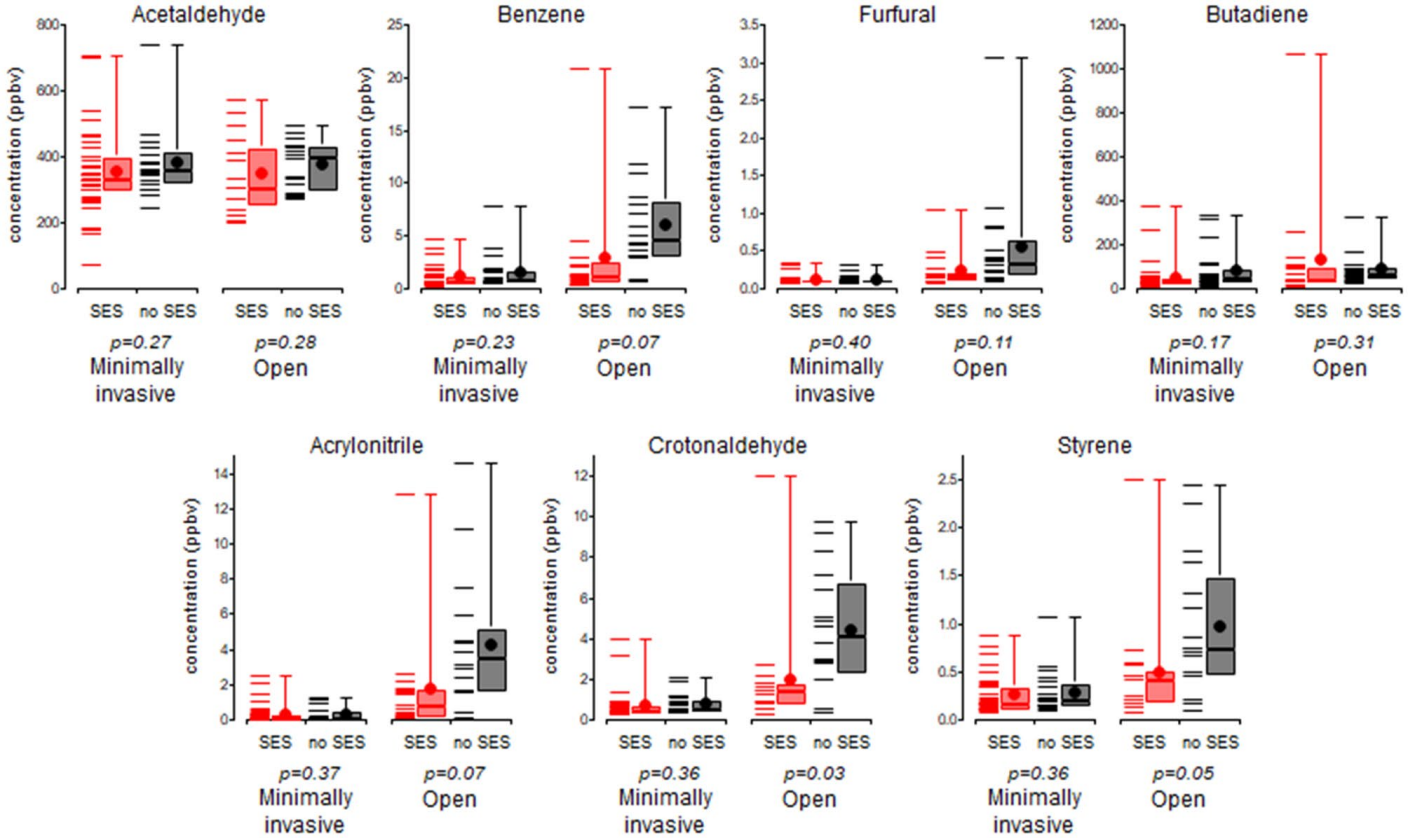
Maximum concentrations of selected VOCs at surgeon level during open procedures and minimally invasive procedures. Each subplot compares operations in which SES was used (red) to operations in which it was not (black). *p *values are given. The horizontal dash marks are the concentrations measured during individual operations, and the box and whisker plots show the maximum, 75th percentile, average (circle marker), 50th percentile, and 25th percentile for all operations.

In Fig. 4 box plots show the comparisons of concentrations of specific harmful VOC’s with and without SES, between open and minimally invasive surgery.

When all operations with SES and without SES were compared, regardless of measurement location or procedure type, the difference in total VOC was not significant. The average maximum total VOC concentration with SES was 1633 ppb (± 1382), compared to 1716 ppb (± 1049) without SES (*p* = 0.37), and the typical mean total VOC concentration with SES was 381 ppb (± 142) compared to 421 ppb (± 179) without SES (*p* = 0.17). Use of SES reduced both average and maximum experienced concentrations of certain VOCs, but not for all VOCs. VOCs whose mean concentration was lower when an SES was used included acrylonitrile (7 ppb with SES vs 15 ppb without SES, *p* = 0.02), pentadiene (2.4 ppb vs. 2.8 ppb, *p* = 0.02), and methyl-2-butenal (82 ppb vs. 100 ppb, *p* = 0.04). Additionally, maximum concentrations of some additional VOCs were lower when an SES was used, including formaldehyde (1722 ppb vs. 519 ppb, *p* < 0.0001), acetonitrile (8.2 ppb vs. 12.9 ppb, *p* = 0.02), propionitrile (0.6 ppb vs. 1.5 ppb, p = 0.04), crotonaldehyde (0.9 ppb vs. 1.8 ppb, *p* = 0.03) and pyridine (0.12 ppb vs. 0.27 ppb, *p* = 0.04).

The maximum total VOC did not statistically differ between open procedures (on average 2064 ± 1536 ppb) and minimally invasive procedures (1590 ± 1157 ppb) (*p* = 0.10), nor was the mean total VOC significantly different (385 ± 156 ppb in open procedures vs. 415 ± 182 ppb in minimally invasive procedures, *p* = 0.26). The linear regression analysis indicated a similar effect, with maximum total VOC for open procedures about 170 ppb higher than for minimally invasive procedures (mean total VOC, n.s.).

However, some specific VOC levels were increased in open procedures compared to minimally invasive procedures. Nearly all harmful VOCs (Table 1) had statistically significant (all *p* ≤ 0.05) higher maxima during open procedures. Mean concentrations of some distinct VOCs were also increased during open procedures, notably pentadiene (3.4 ppb open vs. 2.57 ppb minimally invasive surgery, *p* = 2 × 10^–5^), crotonaldehyde (0.43 vs. 0.32 ppb, *p* = 4 × 10^–4^), and g-butyrolactone (0.67 vs. 0.52 ppb, *p* = 0.001). Conversely, mean concentrations of acetonitrile (0.90 ppb vs. 1.27 ppb, *p* = 0.004) and acetaldehyde (123 vs. 144 ppb, *p* = 0.04) were increased during minimally invasive procedures.

Since average and total VOCs were different in minimally invasive compared to open surgery, and measurements at surgeon level may be most relevant for understanding related health effects, we performed an in-depth analysis comparing concentrations of specific VOCs at surgeon level, with and without SES, for open surgeries, and separately for minimally invasive surgeries (Fig. 4). The use of a smoke evacuation system reduced exposure of surgeons to harmful compounds during open surgeries, but had less effect in minimally invasive surgeries. For complete data on concentrations of all VOCs with and without SES and surgical procedures, please refer to the Supplement.

In general, we observed that average VOC concentrations at surgeon level are only slightly higher than concentrations in room air, but maximum concentrations at surgeon level can be higher than in room air. This was observed for total VOC and individual harmful VOCs. The average maximum total VOC for all operations measured at surgeon level was 1759 ppb (± 1316), compared to an average maximum of 1442 ppb (± 1051) for all room air measurements (*p* = 0.15). The difference in mean value was 404 ppb (± 172) at surgeon level, compared to 382 ppb (± 120) in room air (*p* = 0.29). The linear regression analysis, which separates the conflating effects of SES use and procedure type, indicates that moving from room air to the location of the surgeon increases the maximum total VOC by about 120 ppb, and the mean total VOC by about 22 ppb. Individual VOCs with the most significant differences between the operating table and room air include hydrogen cyanide (0.46 ppb mean (surgeon level) vs. 0.17 ppb (room air), *p* = 0.0002), formaldehyde (7.9 ppb vs. 3.2 ppb, *p* = 0.0004), butadiene (13.2 ppb vs. 7.5 ppb, *p* = 0.002), butenes (3.8 ppb vs. 1.5 ppb, *p* = 0.0003), and pentadiene (2.9 ppb vs. 1.8 ppb, *p* ~ 0).

### Inhalation exposure of health care professionals

For acetaldehyde, an average concentration of 136 ppb (± 52 ppb SD) was measured, indicating 146 µg (± 56) of acetaldehyde inhaled per operation. Other hazardous compounds included butadiene 15 µg per operation (± 17), formaldehyde 4.8 µg (± 7.3), methanol 4.6 µg (± 3.9), ethanol 34 µg (± 33), phenol 6.3 µg (± 1.3), and butenes 4.3 µg (± 6.6) (see Table 1 for full list). Since inhaled and exhaled breaths were measured separately, the uptake is calculated as the difference in concentration between inhaled and exhaled breath (supplementary Fig. 3). The VOC with the highest concentration difference (largest uptake) was acetaldehyde, for which 77 ppb or about 40% is inhaled per breath. Butadiene and ethanol showed high uptakes as well (3 ppb and 16.9 ppb, respectively).

In general, uptake ranged from 1% (phenol, which is essentially unchanged) to 90% (butadiene), and up to 100% (indane/methylstyrene).

### Variability of VOCs over time

We investigated the temporal evolution of VOC concentrations and observed that maximum concentrations of VOCs are typically experienced within the first few minutes of an operation. To show this, we selected the top 100 VOCs with the highest concentrations for each operation, then determined the time point when each VOC reached the highest concentration (supplementary Fig. 4a; dashed line indicates the expected distribution if there were no systematic patterns). Maximum concentrations of particular VOCs (e.g., acetaldehyde) are most likely to be detected within the first 5–10 min of an operation. However, some VOCs are more likely to reach a maximum later (supplementary Fig. 4b).

## Discussion

Here we show that health care professionals are exposed to considerable levels of irritating, toxic, carcinogenic and mutagenic volatile organic compounds when electrocautery devices are used in the OR. Exposure is not limited to the operating team at the OR table, but extends to all staff present in the OR (including, for example, nurses and anesthetists). Choice of operative technique and use of hand-held SES may reduce maximum concentrations of specific hazardous substances, especially in open surgery.

When considering concentrations measured and their relationship to exposure limits defined by the National Institute for Occupational Safety and Health (NIOSH) and earlier guidelines (OSHA)^10^, it appears that most limits are based on Threshold Limit Values (TLVs) from 1968^11^. It is important to note, however, that limits are generally established for individual compounds and do not take into account exposure to mixtures of toxic and carcinogenic substances and their corresponding cumulative effects. In addition, exposure limits for many substances have not yet been defined, and there is exposure to various compounds with direct genotoxicity (e.g., formaldehyde, acetaldehyde, butadiene etc.), regardless of the concentration^12^.

When we examined the levels of compounds in electrocautery smoke, we found many similarities to compounds commonly found in cigarette smoke (e.g., acetaldehyde at around 2000 µg per cigarette and butadiene at 130 µg per cigarette^13,14^). Our data show that concentrations measured during inspiratory/expiratory breath analyses are comparable to exposure to about one tenth of an unfiltered cigarette per surgical procedure. Similar to cigarette smoking, the risk of OR electrocautery smoke may likely accumulate, resulting in health problems over time.

Important limitations of this study warrant discussion. First, we present data from a monocentric investigation, limiting external validity. Nevertheless, randomization of the use of SES may have enabled estimation of SES-induced effects. Second, different types of electrocautery devices are available, just as there are different hand-held SES devices, and thus different VOC concentrations may be generated. Nevertheless, the study was deliberately designed to assess real-life conditions, using a stable OR set-up, rather than artificial trial conditions. In addition we have investigated the effectiveness of the SES-system used for this very study and have found that the construction with on-tip smoke evacuation on the cautery device is amongst the most practical and effective^15^.

Also, it is important to state that our study only focused on thoracic surgical procedures, during which mostly the access to the operative field causes electrocautery smoke emissions. The findings and results from other, more ‘electrocautery-intense’ surgeries (i.e. major soft tissue procedures such as for example abdominoplasty or dermolipectomy) might cause even more air pollution in the OR.

Other groups have investigated the issue of surgical smoke in other settings, for example during Tonsillectomy^16^. Although this specific surgical procedure seems to cause lower concentrations of hazardous VOC’s overall, the use of a smoke-evacuator pencil cautery still significantly reduced the smoke exposure. While the former is likely due to the relatively ‘small procedure’ (in terms of wound area and operating time), the latter might be the explained by an operation taking place in a relatively small and more or less contained space.

Cheng et al.^17^ on the other hand found quite high concentrations of harmful VOC’s during breast surgery, and were furthermore able to demonstrate that a higher electrocautery power (> 27.5 watts) would produce higher concentrations of VOC’s. Similarly, Tokuda et al.^18^ investigated hazardous electrocautery fumes during breast surgeries and found, in line with our own findings, that the difference to the cautery source does not result in significantly lower concentrations of harmful VOC’s and that in fact all OR personnel is exposed to said substances. In contrast to our study results, the Tokuda group found a reduction of total VOC’s when an SES was used, which might be related to the specific type of operations with predominantly cauterization of fatty tissue during breast-conserving surgery and mastectomy procedures. Again similarly as in our study, one of the main harmful substances, Formaldehyde, was significantly reduced by the SES (Tokuda: significant reduction of average concentrations; Our study: significant reduction of maximum concentrations).

In summary our data indicate significant exposure of health care professionals to harmful volatile organic compounds in the OR. While the use of an SES, the type of surgical approach, and the distance to the cautery source did not have a significant impact on total VOC’s, all 3 factors at least resulted in a reduction of maximum concentrations of specific harmful VOC’s. We therefore recommend reducing maximum concentrations of harmful VOC’s whenever possible, until more effective measures have been investigated and developed to protect health care personnel from electrocautery smoke.

## Supplementary Information

Supplementary Information.
