# Supplementary material for: Electrocautery smoke exposure and efficacy of smoke evacuation systems in minimally invasive and open surgery: a prospective randomized study
Source: Sci Rep. 2022 Mar 23;12:4941. doi: 10.1038/s41598-022-08970-y (PMC8943181; doi:10.1038/s41598-022-08970-y)
Supplement: Supplementary file 1 — Supplementary Information. [file 41598_2022_8970_MOESM1_ESM.docx]

**Supplemental Information** for: Electrocautery smoke exposure and efficacy of smoke evacuation systems in minimally invasive and open surgery: a prospective randomized study

Gregor J. Kocher^1*^, MD; Abigail R. Koss^2^, BS, PhD; Michael Groessl^3^, PhD; Joerg C. Schefold^4^, MD; Markus M. Luedi^5^, MD; Christopher Quapp^1^, MD; Patrick Dorn^1^, MD; Jon Lutz^1^, MD; Luca Cappellin^2^, MSc, PhD; Manuel Hutterli^2^, PhD; Felipe D. Lopez-Hilfiker^2^, PhD; Mohammad Al-Hurani^1,6^, MD, and Sergio B. Sesia^1^, MD

^1^ Division of General Thoracic Surgery, Inselspital, Bern University Hospital, University of Bern, Bern, Switzerland

^2^ Tofwerk AG, Thun, Switzerland

^3^ Department of Nephrology and Hypertension and Department of BioMedical Research, Inselspital, Bern University Hospital, University of Bern, Bern, Switzerland

^4^ Department of Intensive Care Medicine, Inselspital, Bern University Hospital, University of Bern, Bern, Switzerland

^5^ Department of Anesthesiology and Pain Medicine, Inselspital, Bern University Hospital, University of Bern, Bern, Switzerland

^6^ Department of General and Special Surgery, Faculty of Medicine, The Hashemite University, Zarqa, Jordan

*corresponding author:

Gregor J. Kocher, MD, MBA, Professor of Thoracic Surgery

Division of General Thoracic Surgery, Bern University Hospital

CH-3010 Bern, Switzerland, Tel.: +41 31 632 04 71, E-mail: [gregor.kocher@hin.ch](mailto:gregor.kocher@hin.ch)

# Supplementary Figure 1. Typical concentration of total VOC measured during operations.


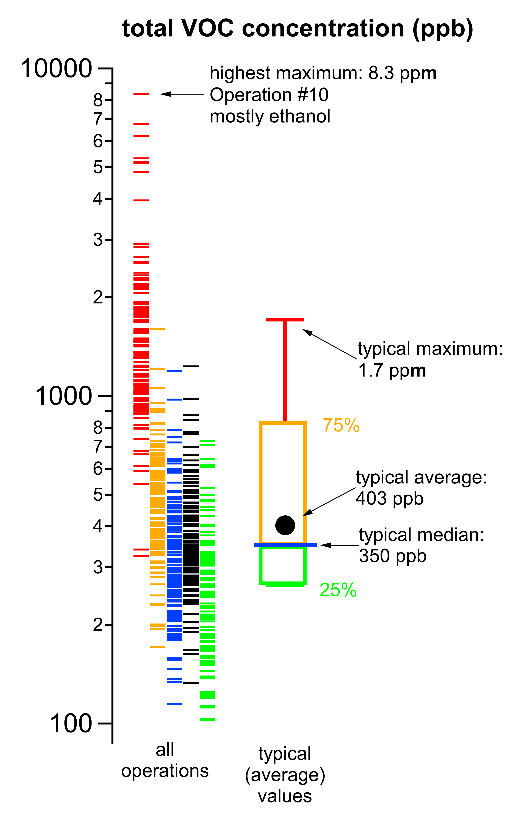


# Supplementary Figure 2. Average mass spectrum during all operations. Major peaks are noted with their likely VOC identity. Mass-to-charge ratios larger than 210 were measured, but had small signal and are not shown in this figure.


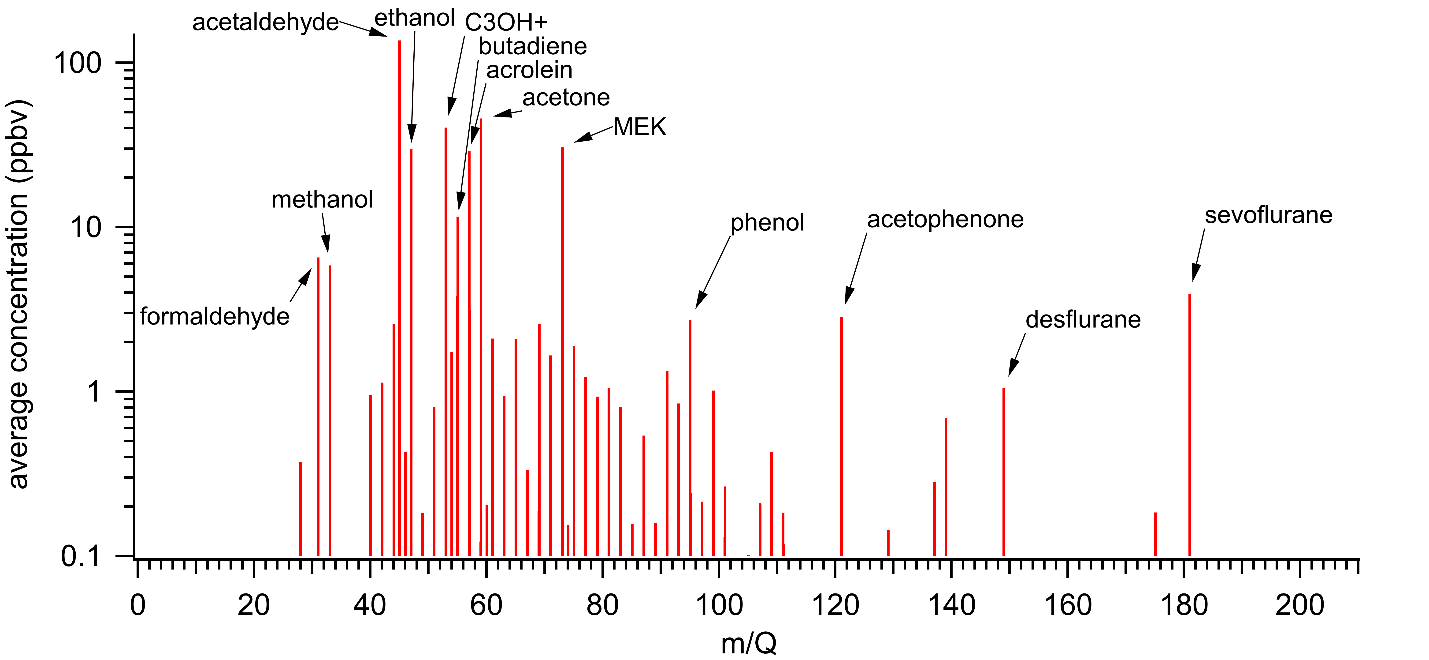


# Supplementary Figure 3. Breath experiment mass spectrum showing the difference in concentration between inhaled and exhaled breath. VOCs with higher concentration in inhaled breath are shown with positive values, in blue, and VOCs with higher concentration in exhaled breath are shown with negative values, in red.


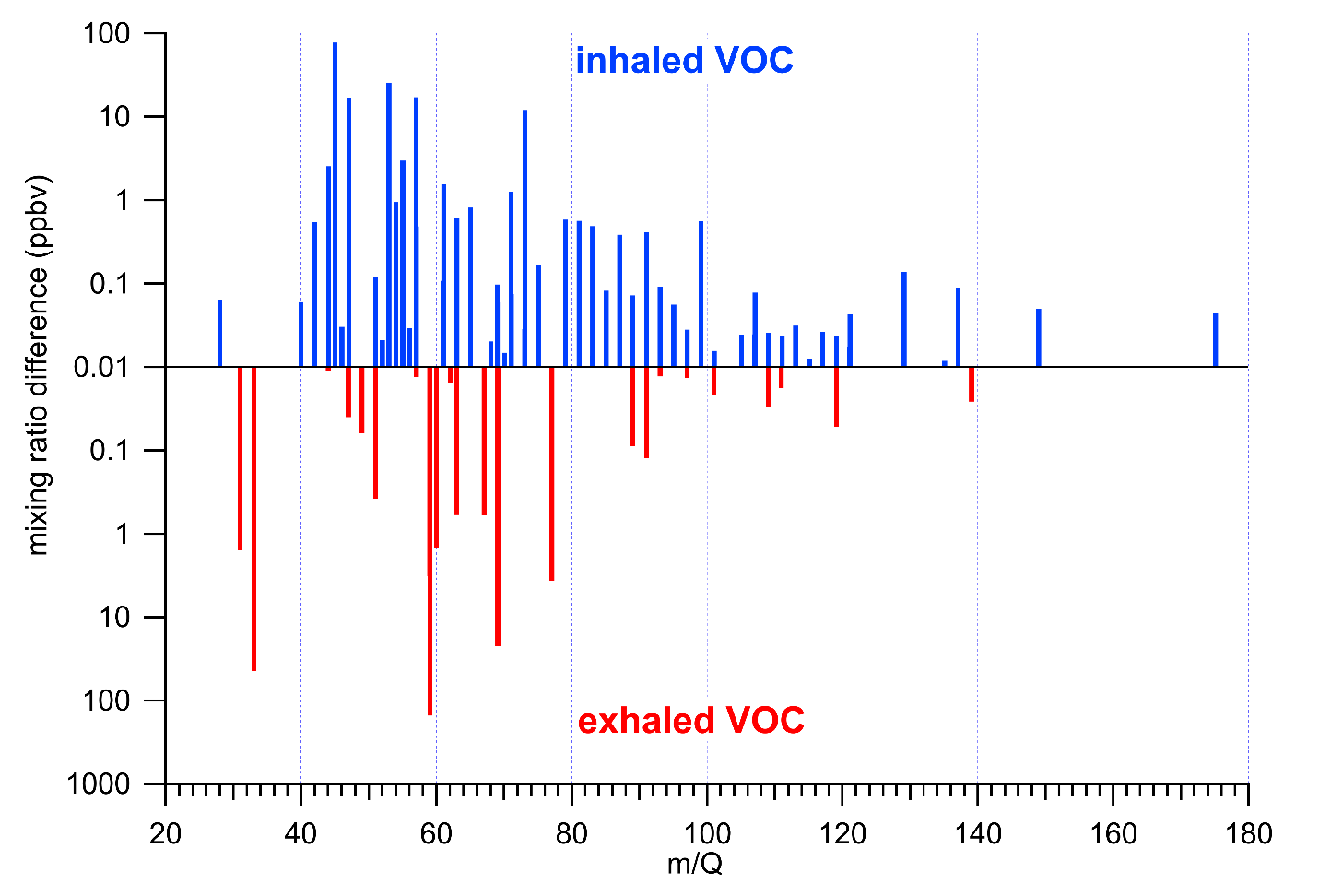


# Supplementary Figure 4. (A) Time of VOC maximum for each of the 100 most abundant VOCs during each operation. (B) Time of maximum concentration for acetaldehyde, acetone, benzene, and sevoflurane.

#
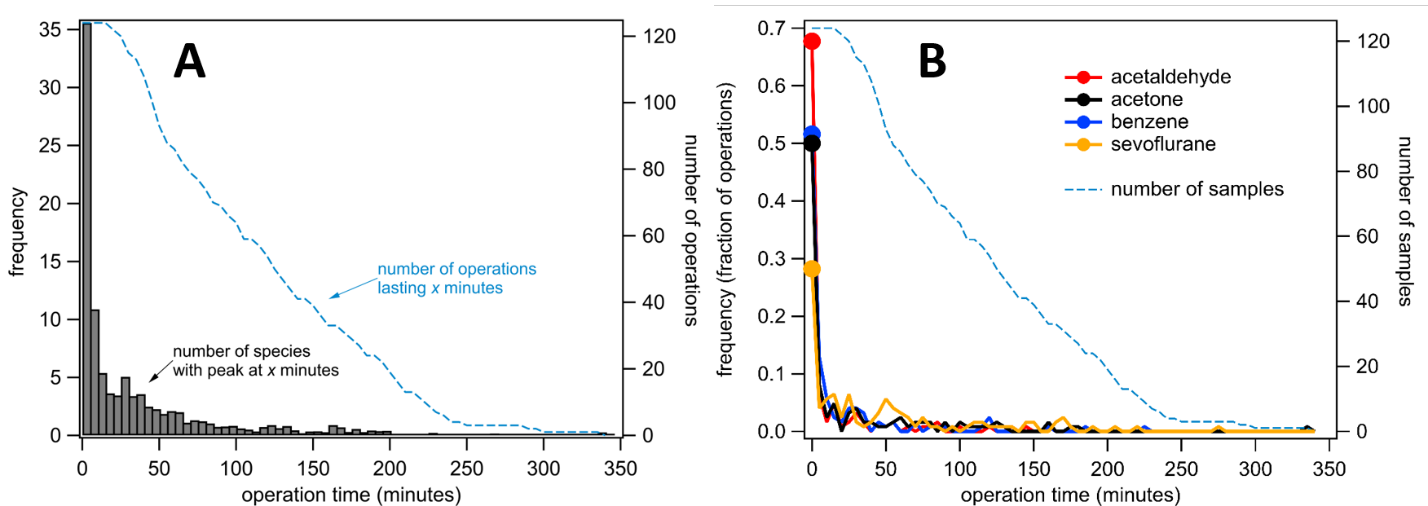


Supplementary Figures 5. Box plot comparisons of experimental parameters.


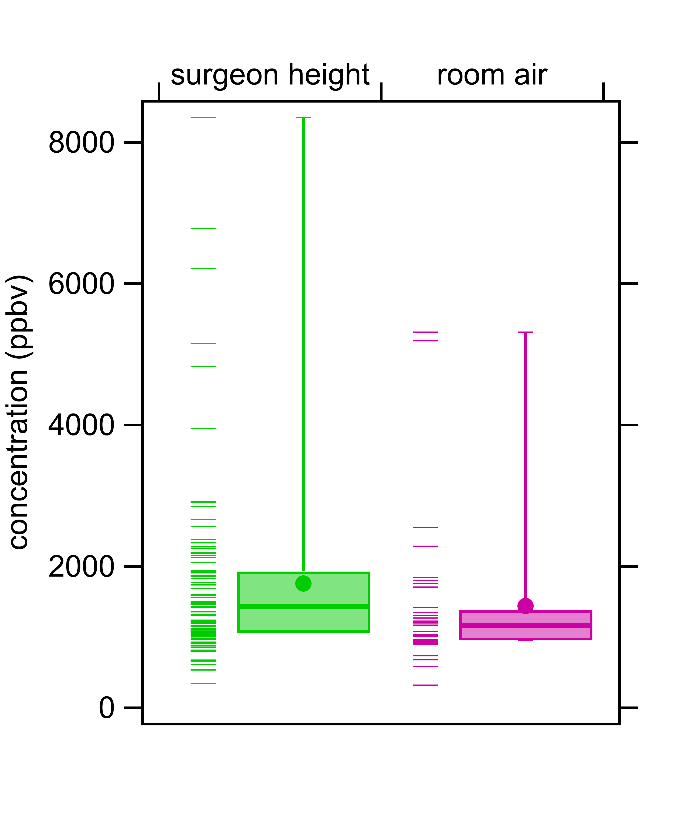
SF5.1. Comparison of measurement at surgeon height and in room air. Total VOC, maximum concentrations:

SF 5.2. Comparison of measurement at surgeon height and in room air.

Total VOC, mean concentrations:


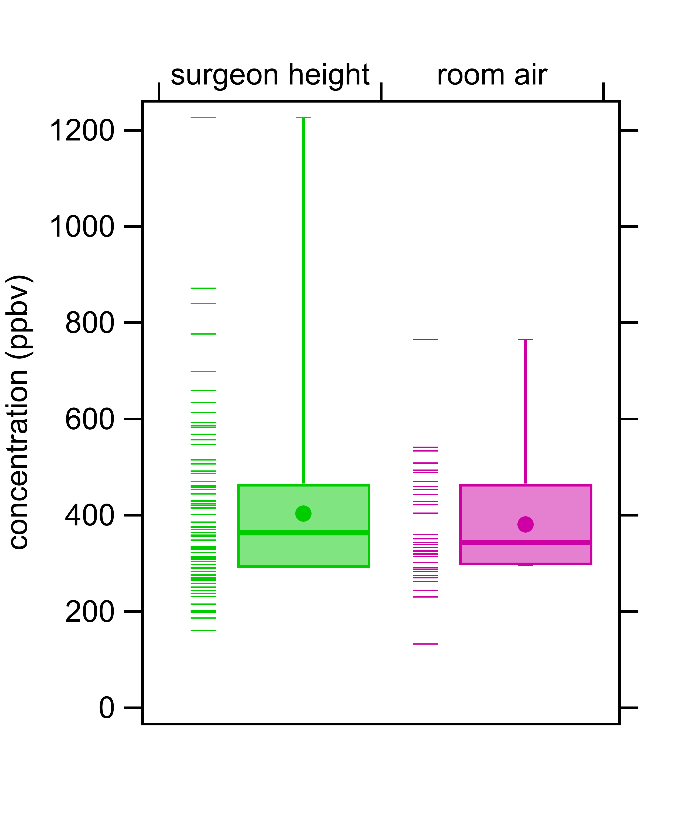


SF 5.3. Comparison of measurement at surgeon height and in room air. Specific harmful VOCs, maximum concentrations:
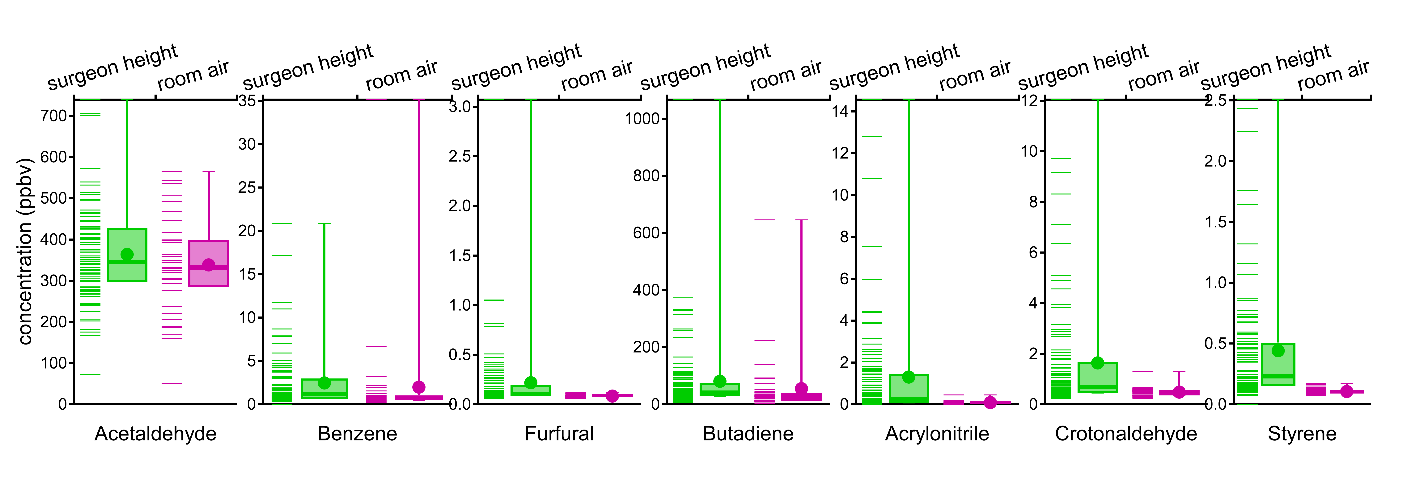


SF 5.4. Comparison of measurement at surgeon height and in room air.

Specific harmful VOCs, mean concentrations:


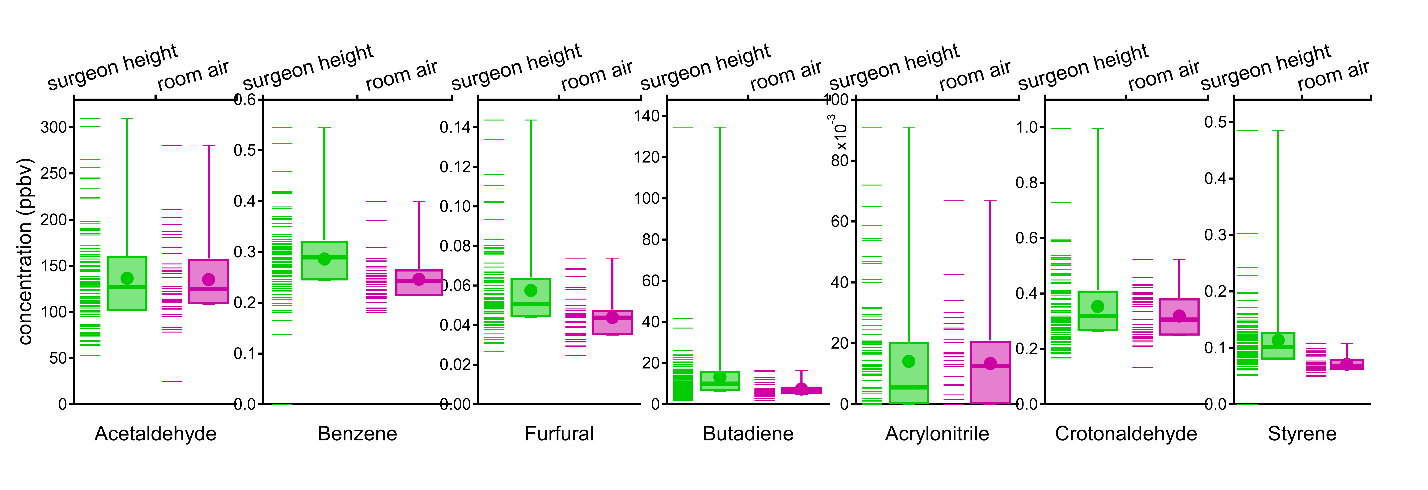


## SF 6.1. Comparison of operations with and without SES. Total VOC, maximum concentrations:


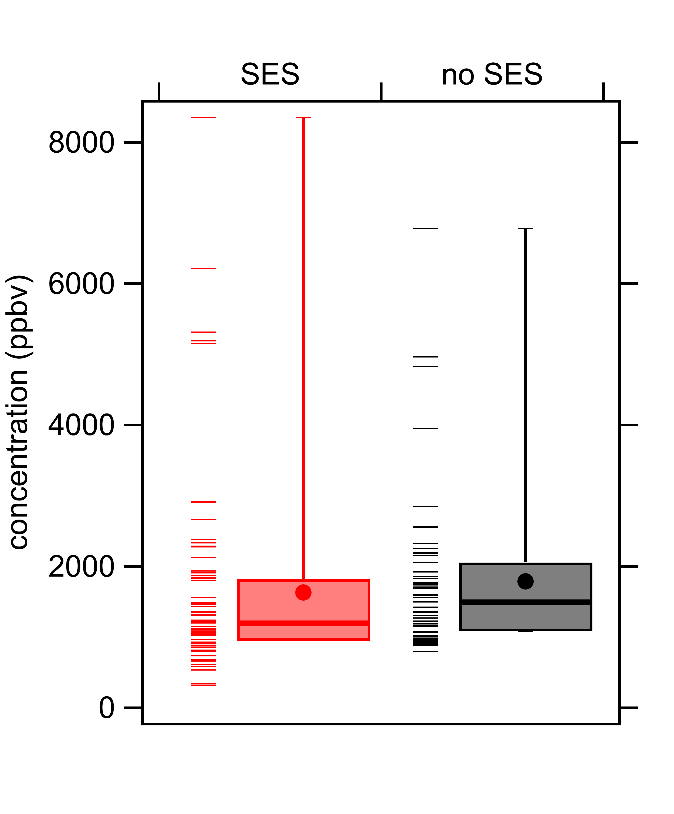


SF 6.2. Comparison of operations with and without SES. Total VOC, mean concentrations:
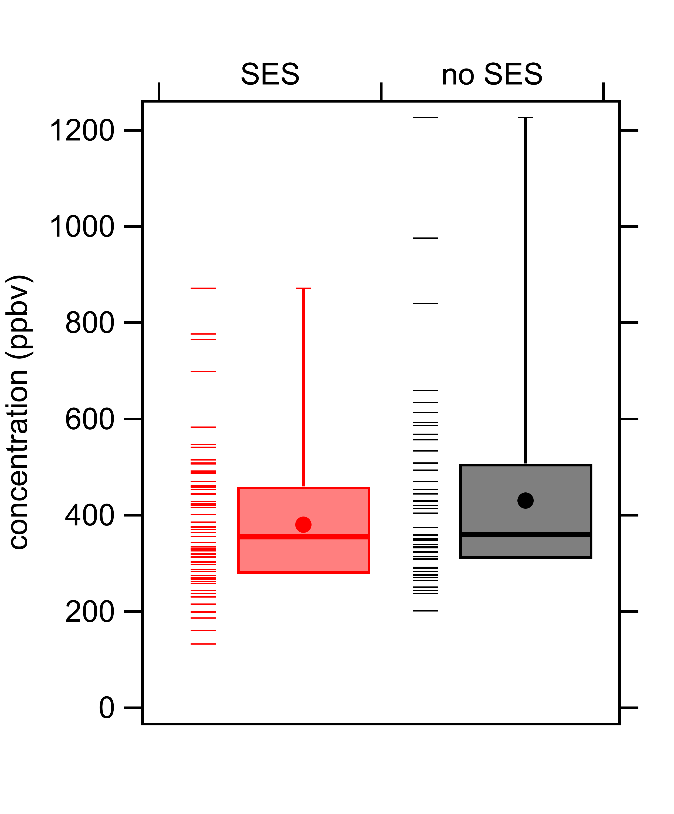


SF 6.3. Comparison of operations with and without SES. Specific harmful VOCs, maximum concentrations:


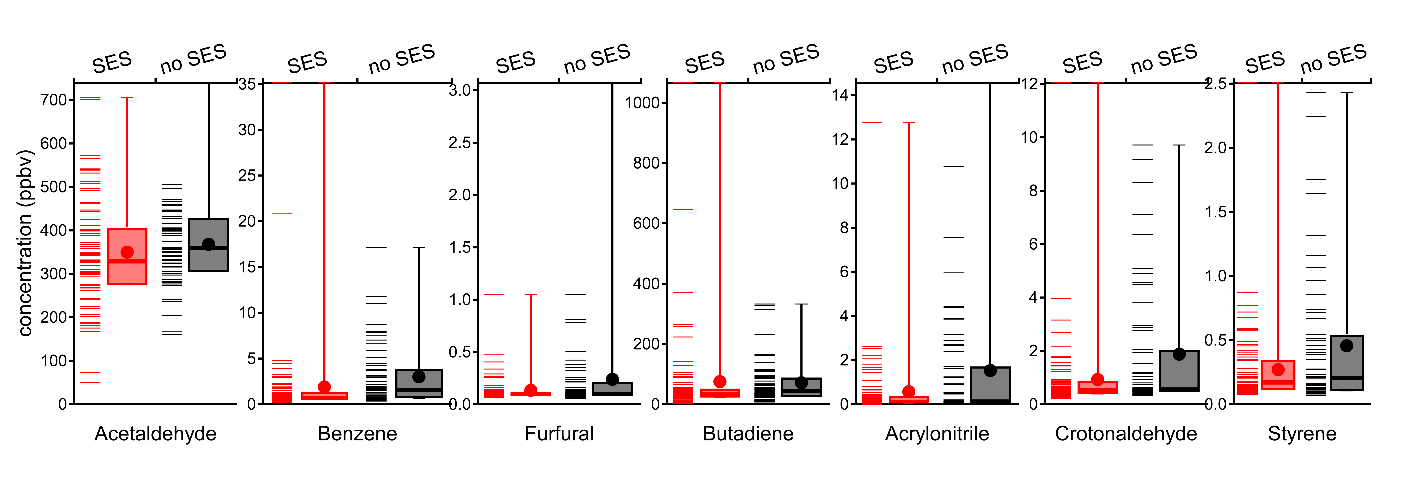


SF 6.4. Comparison of operations with and without SES. Specific harmful VOCs, mean concentrations:


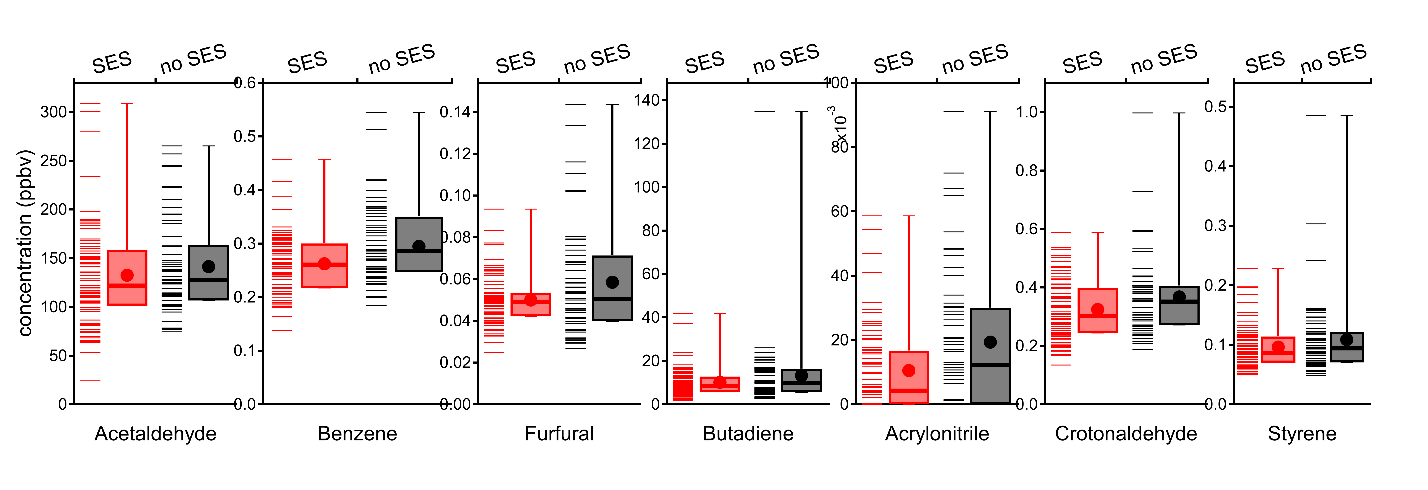


## S7.1. Comparison of minimally invasive and open operations. Total VOC, maximum concentrations:


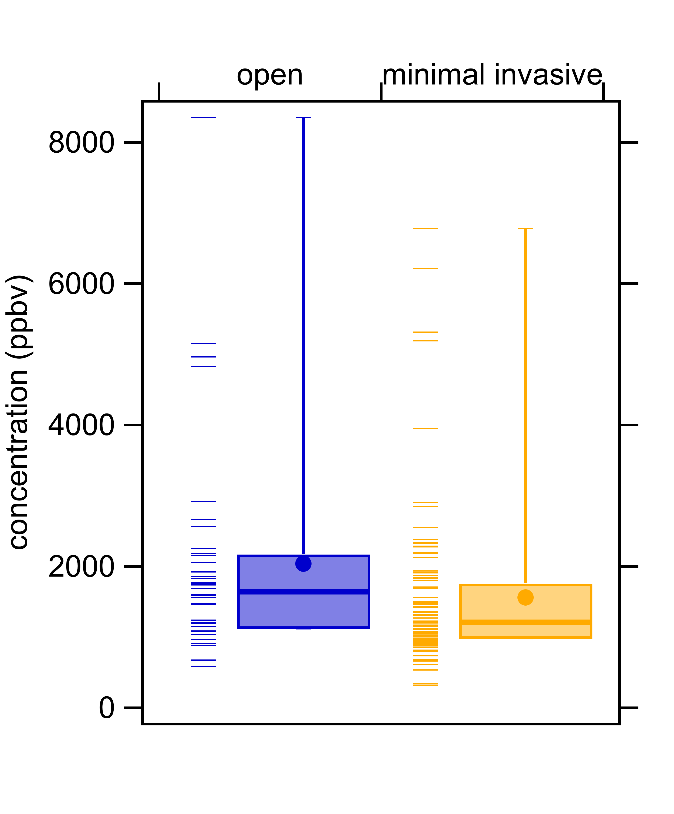


S7.2. Comparison of minimally invasive and open operations. Total VOC, mean concentrations:


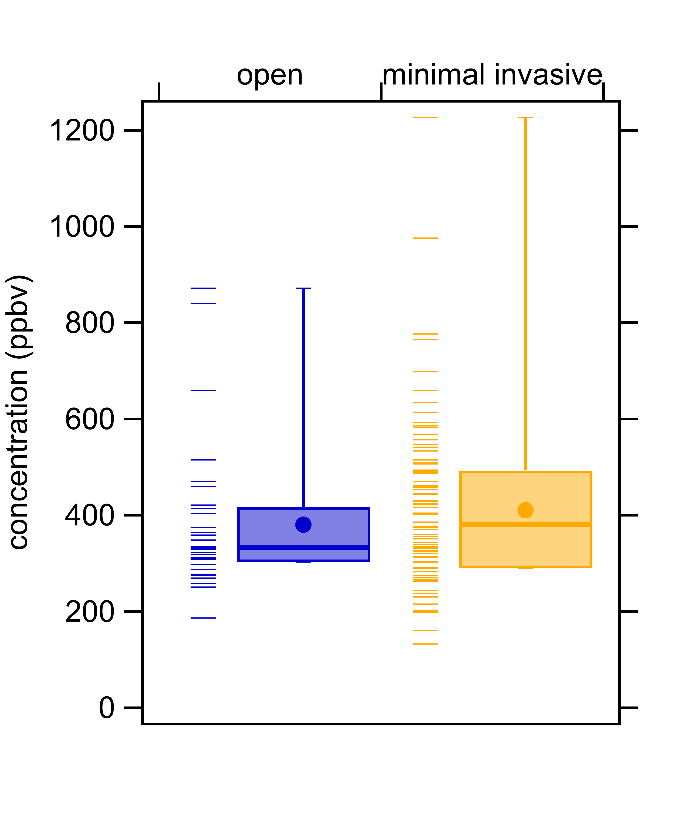


S7.3 Comparison of minimally invasive and open operations. Specific harmful VOCs, maximum concentrations:


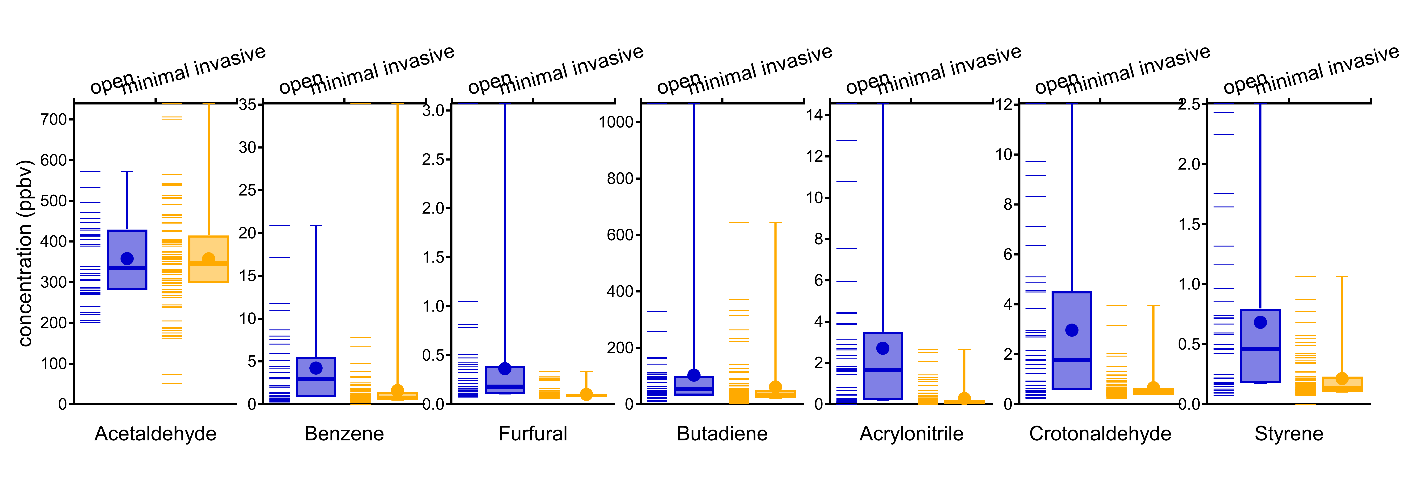


S7.4. Comparison of minimally invasive and open operations. Specific harmful VOCs, maximum concentrations:


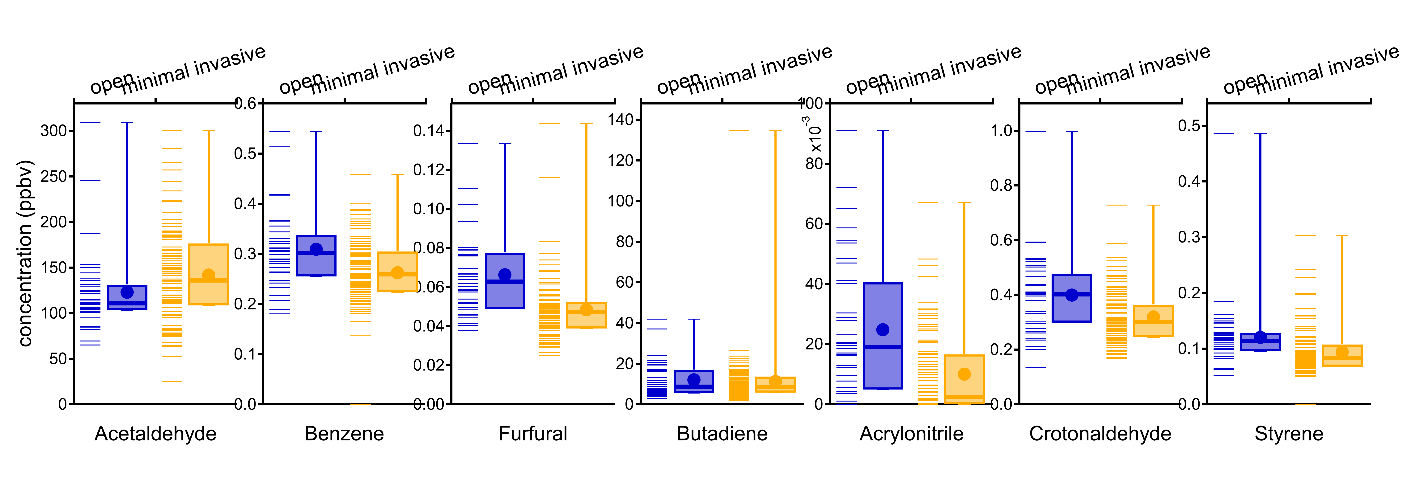


# Supplementary Table 1. Identities and mixing ratios of detected VOC:

| Detected ion formula | Exact mass:charge ratio (Th) | Average detected mixing ratio (ppb) | Maximum detected mixing ratio (ppb) |
| --- | --- | --- | --- |
| HCNH+ | 28.01818 | 0.36 | 22.71 |
| CH2OH+ | 31.01784 | 6.48 | 1721.95 |
| CH4OH+ | 33.03349 | 6.83 | 287.34 |
| C2HNH+ | 40.01817 | 0.97 | 27.52 |
| C2H3NH+ | 42.03383 | 1.13 | 55.38 |
| HNCOH+ | 44.01309 | 0.03 | 2.50 |
| C2H5NH+ | 44.04948 | 2.57 | 93.46 |
| C2H4OH+ | 45.03349 | 134.61 | 739.66 |
| CH3NOH+ | 46.02874 | 0.42 | 7.98 |
| C2H7NH+ | 46.06513 | 0.04 | 0.90 |
| CFO+ | 46.99277 | 0.01 | 4.15 |
| CH2O2H+ | 47.01276 | 0.15 | 19.35 |
| C2H6OH+ | 47.04914 | 30.15 | 2054.08 |
| CH5NOH+ | 48.04439 | 0.04 | 25.55 |
| CH4SH+ | 49.01065 | 0.01 | 7.56 |
| CH4O2H+ | 49.0284 | 0.19 | 130.45 |
| CHF2+ | 51.00408 | 0.74 | 642.79 |
| CH6O2H+ | 51.04406 | 0.02 | 3.34 |
| C3HNH+ | 52.01817 | 0.02 | 5.41 |
| C3OH+ | 53.00219 | 39.85 | 485.87 |
| CH2F2H+ | 53.01973 | 0.16 | 33.85 |
| C4H4H+ | 53.03858 | 0.39 | 38.38 |
| CN3+ | 54.00868 | 1.72 | 24.98 |
| C3H3NH+ | 54.03383 | 0.01 | 14.57 |
| C3H2OH+ | 55.01784 | 3.78 | 46.51 |
| C4H6H+ | 55.05423 | 11.35 | 1066.49 |
| C2HNOH+ | 56.01309 | 0.01 | 0.32 |
| C3H5NH+ | 56.04948 | 0.02 | 13.63 |
| C3H4OH+ | 57.03349 | 29.01 | 660.26 |
| C2H5N2+ | 57.04472 | 0.87 | 48.91 |
| C4H9+ | 57.06988 | 3.12 | 128.66 |
| C2H3NOH+ | 58.02874 | 0.03 | 1.25 |
| C3H7NH+ | 58.06513 | 0.03 | 0.49 |
| C2H2O2H+ | 59.01276 | 0.16 | 308.13 |
| C3H6OH+ | 59.04914 | 47.81 | 521.66 |
| C2H5NOH+ | 60.04439 | 0.21 | 179.41 |
| C3H9NH+ | 60.08078 | 0.03 | 3.91 |
| COSH+ | 60.97426 | <10 ppt | 1.54 |
| C2H4O2H+ | 61.0284 | 2.10 | 705.51 |
| CH4N2OH+ | 61.03964 | 0.04 | 129.74 |
| C3H9O+ | 61.06479 | 1.65 | 253.36 |
| CClNH+ | 61.9792 | <10 ppt | 0.07 |
| CH3NO2H+ | 62.02366 | 0.01 | 0.90 |
| C2H7NOH+ | 62.06004 | 0.01 | 6.16 |
| CClO+ | 62.96322 | <10 ppt | 0.83 |
| H2CO3H+ | 63.00767 | <10 ppt | 0.22 |
| C2H6SH+ | 63.0263 | 0.03 | 3.86 |
| C2H6O2H+ | 63.04406 | 0.94 | 14.38 |
| C4HNH+ | 64.01817 | <10 ppt | 0.04 |
| CH5NO2H+ | 64.03931 | <10 ppt | 0.05 |
| C2H5ClH+ | 65.01525 | <10 ppt | 0.31 |
| CH4O3H+ | 65.02332 | <10 ppt | 0.13 |
| C5H4H+ | 65.03857 | 0.04 | 19.61 |
| C2H8O2H+ | 65.05971 | 2.14 | 242.15 |
| C4H3NH+ | 66.03383 | <10 ppt | 2.12 |
| CF2HO+ | 66.999 | 0.01 | 38.66 |
| C4H2OH+ | 67.01784 | <10 ppt | 0.30 |
| CH6OSH+ | 67.02121 | <10 ppt | 0.16 |
| CH7O3+ | 67.03897 | <10 ppt | 0.84 |
| C5H6H+ | 67.05423 | 0.34 | 36.07 |
| C3HNOH+ | 68.01309 | <10 ppt | 0.07 |
| C4H5NH+ | 68.04948 | 0.01 | 20.49 |
| C3O2H+ | 68.99711 | 0.19 | 17.93 |
| C4H4OH+ | 69.03349 | 0.05 | 5.90 |
| C5H8H+ | 69.06988 | 2.71 | 196.38 |
| C2NO2+ | 69.99236 | <10 ppt | 0.26 |
| C3H3NOH+ | 70.02874 | <10 ppt | 0.86 |
| C4H7NH+ | 70.06513 | 0.02 | 4.52 |
| C3H2O2H+ | 71.01276 | 1.65 | 79.68 |
| C4H6OH+ | 71.04914 | 0.35 | 12.05 |
| C5H10H+ | 71.08553 | 0.36 | 16.65 |
| C2HNO2H+ | 72.008 | <10 ppt | 0.27 |
| C3H5NOH+ | 72.04439 | 0.03 | 1.07 |
| C4H9NH+ | 72.08077 | 0.01 | 0.15 |
| C3H4O2H+ | 73.0284 | 0.23 | 182.94 |
| C4H8OH+ | 73.06479 | 30.52 | 936.97 |
| C2FHNO+ | 74.00367 | <10 ppt | 0.76 |
| C2H3NO2H+ | 74.02366 | 0.01 | 0.16 |
| CH3N3OH+ | 74.03489 | 0.01 | 0.79 |
| C3H7NOH+ | 74.06004 | 0.15 | 78.63 |
| C4H11NH+ | 74.09643 | 0.04 | 9.87 |
| C2H2O3H+ | 75.00767 | 0.02 | 0.44 |
| C3H6SH+ | 75.0263 | 0.09 | 3.54 |
| C3H6O2H+ | 75.04406 | 1.91 | 63.75 |
| C2H5NSH+ | 76.02155 | <10 ppt | 0.98 |
| C2H5NO2H+ | 76.03931 | 0.01 | 0.65 |
| C3H9NOH+ | 76.07569 | <10 ppt | 2.51 |
| C5HO+ | 77.00219 | 0.02 | 0.22 |
| C2H4O3H+ | 77.02332 | 0.03 | 0.18 |
| C6H5+ | 77.03857 | 0.04 | 2.88 |
| C3H8O2H+ | 77.05971 | 1.28 | 154.24 |
| CH4NOS+ | 78.00081 | <10 ppt | 0.06 |
| C5H3NH+ | 78.03383 | 0.01 | 1.14 |
| C3F2H5+ | 79.03539 | 0.02 | 6.78 |
| C6H6H+ | 79.05423 | 0.27 | 35.14 |
| C3H11O2+ | 79.07536 | 0.95 | 140.73 |
| C4HONH+ | 80.01309 | <10 ppt | 0.10 |
| CH5NO3H+ | 80.03422 | <10 ppt | 0.03 |
| C5H5NH+ | 80.04948 | 0.02 | 2.61 |
| C2H2FClH+ | 80.99018 | 0.02 | 0.22 |
| CH4O4H+ | 81.01823 | <10 ppt | 0.10 |
| C5H4OH+ | 81.03349 | 0.04 | 2.78 |
| C4H5N2+ | 81.04472 | 0.02 | 2.92 |
| C6H8H+ | 81.06988 | 1.03 | 64.85 |
| C4H3NOH+ | 82.02874 | <10 ppt | 0.05 |
| C5H7NH+ | 82.06513 | 0.01 | 4.00 |
| CCl2H+ | 82.94498 | <10 ppt | 0.17 |
| C4H2O2H+ | 83.01276 | 0.01 | 0.09 |
| CH7O4+ | 83.03388 | <10 ppt | 0.32 |
| C5H6OH+ | 83.04914 | 0.10 | 4.19 |
| C4H6N2H+ | 83.06037 | 0.02 | 8.74 |
| C6H10H+ | 83.08553 | 0.80 | 146.68 |
| C3HNO2H+ | 84.008 | <10 ppt | 0.01 |
| C4H5NOH+ | 84.04439 | <10 ppt | 1.42 |
| C5H9NH+ | 84.08077 | 0.01 | 5.30 |
| CClF2+ | 84.96511 | <10 ppt | 0.54 |
| C2N2SH+ | 84.9855 | <10 ppt | 0.04 |
| C4H4O2H+ | 85.0284 | 0.13 | 3.04 |
| C5H8OH+ | 85.06479 | 0.09 | 2.96 |
| C6H12H+ | 85.10117 | 0.16 | 70.55 |
| C3H4NS+ | 86.0059 | <10 ppt | 0.10 |
| C3H3NO2H+ | 86.02366 | <10 ppt | 0.03 |
| C4H7NOH+ | 86.06004 | 0.02 | 0.55 |
| C5H11NH+ | 86.09643 | <10 ppt | 0.24 |
| CHClF2H+ | 86.98076 | <10 ppt | 0.11 |
| C3H2O3H+ | 87.00767 | 0.02 | 0.18 |
| C4H6O2H+ | 87.04406 | 0.54 | 4.34 |
| C5H10OH+ | 87.08044 | 0.52 | 128.95 |
| C2HNO3H+ | 88.00292 | <10 ppt | 0.02 |
| C3H6NO2+ | 88.03931 | 0.02 | 0.14 |
| C4H9NOH+ | 88.07569 | 0.03 | 0.52 |
| C5H13NH+ | 88.11208 | <10 ppt | 0.04 |
| C3H4O3H+ | 89.02332 | 0.13 | 5.31 |
| C2H4N2O2H+ | 89.03455 | 0.01 | 2.95 |
| C4H8O2H+ | 89.05971 | 0.16 | 103.47 |
| C2H3NO3H+ | 90.01857 | 0.01 | 0.07 |
| C3H8NS+ | 90.03719 | <10 ppt | 0.11 |
| C3H7NO2H+ | 90.05496 | 0.01 | 0.16 |
| C4H11NOH+ | 90.09134 | 0.01 | 0.67 |
| C2H3O4+ | 91.00259 | <10 ppt | 2.90 |
| C3H7O3+ | 91.03897 | 0.03 | 0.72 |
| C7H6H+ | 91.05423 | 0.36 | 37.12 |
| C4H11O2+ | 91.07536 | 1.34 | 70.63 |
| C2H5NO3H+ | 92.03422 | <10 ppt | 0.06 |
| C6H6N+ | 92.04948 | 0.01 | 0.86 |
| C4H13NOH+ | 92.10699 | <10 ppt | 0.10 |
| C3H9OS+ | 93.03687 | 0.31 | 1.81 |
| C7H8H+ | 93.06988 | 0.85 | 123.60 |
| C5H3NOH+ | 94.02874 | 0.01 | 0.19 |
| C6H7NH+ | 94.06513 | 0.02 | 0.81 |
| C5H2O2H+ | 95.01276 | 0.09 | 5.03 |
| C6H6OH+ | 95.04914 | 2.71 | 10.44 |
| C3H10O3H+ | 95.07027 | 0.01 | 0.79 |
| C7H10H+ | 95.08553 | 0.24 | 7.45 |
| CH5NO4H+ | 96.02914 | <10 ppt | 0.04 |
| C5H5NOH+ | 96.04439 | 0.02 | 0.32 |
| C6H9NH+ | 96.08077 | 0.01 | 1.23 |
| C2H2Cl2H+ | 96.96063 | 0.01 | 0.09 |
| C5H4O2H+ | 97.0284 | 0.06 | 3.07 |
| C2H8O4H+ | 97.04954 | 0.01 | 0.22 |
| C6H8OH+ | 97.06479 | 0.05 | 1.74 |
| C3H13O3+ | 97.08592 | 0.03 | 2.46 |
| C7H12H+ | 97.10117 | 0.21 | 4.57 |
| C4H3NO2H+ | 98.02366 | <10 ppt | 1.36 |
| C5H7NOH+ | 98.06004 | 0.01 | 0.75 |
| C6H11NH+ | 98.09643 | 0.01 | 1.50 |
| C4H2O3H+ | 99.00767 | 0.03 | 1.33 |
| C5H6O2H+ | 99.04406 | 0.05 | 1.04 |
| C6H10OH+ | 99.08044 | 1.01 | 17.93 |
| C7H15+ | 99.11683 | 0.04 | 0.43 |
| C4H6NS+ | 100.0216 | <10 ppt | 0.26 |
| C4H5NO2H+ | 100.0393 | 0.01 | 0.20 |
| C5H9NOH+ | 100.0757 | 0.02 | 0.25 |
| C6H13NH+ | 100.1121 | <10 ppt | 0.09 |
| CCl2F+ | 100.9356 | 0.01 | 0.09 |
| C4H4O3H+ | 101.0233 | 0.13 | 1.98 |
| C5H8O2H+ | 101.0597 | 0.26 | 2.13 |
| C6H12OH+ | 101.0961 | 0.08 | 24.87 |
| C3H3NO3H+ | 102.0186 | <10 ppt | 0.07 |
| C4H8NO2+ | 102.055 | 0.01 | 0.21 |
| C5H11NOH+ | 102.0913 | 0.03 | 0.63 |
| C6H15NH+ | 102.1277 | <10 ppt | 0.03 |
| C3H2O4H+ | 103.0026 | <10 ppt | 0.02 |
| C2H2F4H+ | 103.0165 | <10 ppt | 0.07 |
| C4H6O3H+ | 103.039 | 0.03 | 7.73 |
| C8H6H+ | 103.0542 | 0.03 | 1.52 |
| C5H10O2H+ | 103.0754 | 0.03 | 11.52 |
| C6H14OH+ | 103.1117 | <10 ppt | 108.03 |
| C3H5NO3H+ | 104.0342 | <10 ppt | 0.06 |
| C7H5NH+ | 104.0495 | <10 ppt | 0.81 |
| C4H9NO2H+ | 104.0706 | <10 ppt | 0.05 |
| C5H13NOH+ | 104.107 | <10 ppt | 1.49 |
| C3H4O4H+ | 105.0182 | 0.01 | 0.25 |
| C7H4OH+ | 105.0335 | 0.02 | 0.19 |
| C4H8O3H+ | 105.0546 | 0.02 | 0.44 |
| C8H8H+ | 105.0699 | 0.10 | 2.51 |
| C5H12O2H+ | 105.091 | 0.02 | 1.59 |
| C2H3O4NH+ | 106.0135 | <10 ppt | 0.02 |
| C6H3NOH+ | 106.0287 | <10 ppt | 0.01 |
| C3H7NO3H+ | 106.0499 | <10 ppt | 0.03 |
| C7H7NH+ | 106.0651 | <10 ppt | 0.23 |
| C4H11NO2H+ | 106.0863 | <10 ppt | 0.02 |
| C5H15NOH+ | 106.1226 | <10 ppt | 0.01 |
| C2H2O5H+ | 106.9975 | <10 ppt | 3.68 |
| C3H6O4H+ | 107.0339 | 0.01 | 0.23 |
| C7H6OH+ | 107.0491 | 0.21 | 3.92 |
| C4H10O3H+ | 107.0703 | 0.02 | 0.44 |
| C8H10H+ | 107.0855 | 0.21 | 2.95 |
| C5H14O2H+ | 107.1067 | 0.02 | 42.94 |
| C2H6NO2S+ | 108.0114 | <10 ppt | 0.08 |
| C2H5NO4H+ | 108.0291 | <10 ppt | 0.03 |
| C6H5NOH+ | 108.0444 | 0.01 | 0.14 |
| C3H9NO3H+ | 108.0655 | <10 ppt | 0.07 |
| C7H9NH+ | 108.0808 | 0.01 | 0.36 |
| C4H13NO2H+ | 108.1019 | <10 ppt | 0.04 |
| C2H4O5H+ | 109.0132 | <10 ppt | 0.75 |
| C6H4O2H+ | 109.0284 | 0.43 | 8.23 |
| C3H8O4H+ | 109.0495 | 0.02 | 1.06 |
| C7H8OH+ | 109.0648 | 0.03 | 2.47 |
| C6H8N2H+ | 109.076 | 0.01 | 0.15 |
| C4H12O3H+ | 109.0859 | 0.02 | 1.70 |
| C8H12H+ | 109.1012 | 0.29 | 15.05 |
| C5H3NO2H+ | 110.0237 | <10 ppt | 0.04 |
| C2H7NO4H+ | 110.0448 | <10 ppt | 0.08 |
| C6H7NOH+ | 110.06 | <10 ppt | 0.34 |
| C7H11NH+ | 110.0964 | 0.01 | 0.42 |
| C6H3ClH+ | 110.9996 | <10 ppt | 1.59 |
| C5H2O3H+ | 111.0077 | <10 ppt | 1.16 |
| C6H6SH+ | 111.0263 | <10 ppt | 0.17 |
| C6H6O2H+ | 111.0441 | 0.18 | 2.17 |
| C3H10O4H+ | 111.0652 | 0.01 | 0.24 |
| C7H10OH+ | 111.0804 | 0.04 | 0.68 |
| C8H14H+ | 111.1168 | 0.12 | 7.83 |
| C5H5NO2H+ | 112.0393 | <10 ppt | 4.30 |
| C6H9NOH+ | 112.0757 | 0.01 | 1.12 |
| C7H13NH+ | 112.1121 | <10 ppt | 0.20 |
| C3HN2O3+ | 112.9982 | 0.02 | 29.44 |
| C6H5ClH+ | 113.0153 | <10 ppt | 18.08 |
| C5H4O3H+ | 113.0233 | 0.05 | 0.44 |
| C6H8SH+ | 113.042 | 0.01 | 0.51 |
| C6H8O2H+ | 113.0597 | 0.08 | 7.46 |
| C7H12OH+ | 113.0961 | 0.03 | 1.07 |
| C8H16H+ | 113.1325 | 0.01 | 0.14 |
| C4H3NO3H+ | 114.0186 | 0.01 | 0.35 |
| C5H8NS+ | 114.0372 | <10 ppt | 0.03 |
| C5H7NO2H+ | 114.055 | <10 ppt | 0.31 |
| C6H11NOH+ | 114.0913 | 0.08 | 1.05 |
| C7H15NH+ | 114.1277 | <10 ppt | 0.07 |
| C4H2O4H+ | 115.0026 | 0.01 | 0.87 |
| C5H6O3H+ | 115.039 | 0.02 | 0.34 |
| C6H10O2H+ | 115.0754 | 0.08 | 0.96 |
| C7H14OH+ | 115.1117 | 0.06 | 0.78 |
| C8H18H+ | 115.1481 | <10 ppt | 0.06 |
| C4H5NO3H+ | 116.0342 | <10 ppt | 0.18 |
| C5H10NS+ | 116.0529 | <10 ppt | 0.13 |
| C5H9NO2H+ | 116.0706 | <10 ppt | 0.12 |
| C6H13NOH+ | 116.107 | <10 ppt | 0.32 |
| C7H17NH+ | 116.1434 | <10 ppt | 0.01 |
| CCl3+ | 116.906 | 0.04 | 0.14 |
| C2H3Cl2FH+ | 116.9669 | <10 ppt | 0.19 |
| C2HClF3+ | 116.9713 | <10 ppt | 3.23 |
| C4H4O4H+ | 117.0182 | <10 ppt | 8.81 |
| C5H8O3H+ | 117.0546 | 0.01 | 0.15 |
| C9H8H+ | 117.0699 | 0.01 | 0.78 |
| C6H12O2H+ | 117.091 | 0.06 | 5.61 |
| C7H16OH+ | 117.1274 | <10 ppt | 0.08 |
| C3H3NO4H+ | 118.0135 | <10 ppt | 0.03 |
| C4H7NO3H+ | 118.0499 | <10 ppt | 0.05 |
| C8H7NH+ | 118.0651 | <10 ppt | 0.51 |
| C5H11NO2H+ | 118.0863 | <10 ppt | 0.06 |
| C6H15NOH+ | 118.1226 | <10 ppt | 0.01 |
| CHCl3H+ | 118.9217 | <10 ppt | 0.05 |
| C2F5+ | 118.9915 | <10 ppt | 0.08 |
| C3H2O5H+ | 118.9975 | <10 ppt | 1.23 |
| C4H6O4H+ | 119.0339 | <10 ppt | 0.71 |
| C8H6OH+ | 119.0491 | 0.04 | 0.24 |
| C7H6N2H+ | 119.0604 | <10 ppt | 0.07 |
| C5H10O3H+ | 119.0703 | 0.01 | 0.16 |
| C9H10H+ | 119.0855 | 0.07 | 1.48 |
| C6H14SH+ | 119.0889 | <10 ppt | 0.50 |
| C6H14O2H+ | 119.1067 | 0.03 | 25.21 |
| C3H5NO4H+ | 120.0291 | <10 ppt | 0.03 |
| C7H5NOH+ | 120.0444 | <10 ppt | 0.03 |
| C4H9NO3H+ | 120.0655 | <10 ppt | 0.03 |
| C8H9NH+ | 120.0808 | <10 ppt | 0.11 |
| C5H13NO2H+ | 120.1019 | <10 ppt | 0.03 |
| C6H17NOH+ | 120.1383 | <10 ppt | 0.01 |
| CF2Cl2H+ | 120.9418 | <10 ppt | 0.02 |
| C3H4O5H+ | 121.0132 | <10 ppt | 0.39 |
| C4H8SO2H+ | 121.0318 | <10 ppt | 0.92 |
| C4H8O4H+ | 121.0495 | 0.08 | 1.58 |
| C8H8OH+ | 121.0648 | 2.83 | 7.86 |
| C5H12O3H+ | 121.0859 | 0.02 | 14.86 |
| C9H12H+ | 121.1012 | 0.13 | 24.97 |
| C6H16O2H+ | 121.1223 | 0.05 | 140.52 |
| C6H3O2NH+ | 122.0237 | <10 ppt | 0.02 |
| C3H7NO4H+ | 122.0448 | <10 ppt | 0.04 |
| C7H7NOH+ | 122.06 | 0.02 | 0.17 |
| C4H11NO3H+ | 122.0812 | 0.01 | 0.18 |
| C8H11NH+ | 122.0964 | <10 ppt | 0.14 |
| C5H15NO2H+ | 122.1176 | <10 ppt | 9.07 |
| CH3N2O5+ | 123.0037 | <10 ppt | 0.06 |
| C3H6O5H+ | 123.0288 | <10 ppt | 0.09 |
| C7H6O2H+ | 123.0441 | 0.05 | 0.27 |
| C4H10O4H+ | 123.0652 | <10 ppt | 0.06 |
| C8H10OH+ | 123.0804 | 0.01 | 0.58 |
| C7H10N2H+ | 123.0917 | <10 ppt | 0.03 |
| C5H14O3H+ | 123.1016 | <10 ppt | 0.22 |
| C9H14H+ | 123.1168 | 0.07 | 1.22 |
| C6H5NO2H+ | 124.0393 | <10 ppt | 0.13 |
| C3H9NO4H+ | 124.0604 | <10 ppt | 0.02 |
| C7H9NOH+ | 124.0757 | <10 ppt | 0.09 |
| C8H13NH+ | 124.1121 | <10 ppt | 0.19 |
| C6H4O3H+ | 125.0233 | 0.06 | 3.51 |
| C7H8SH+ | 125.042 | 0.01 | 0.23 |
| C7H8O2H+ | 125.0597 | 0.02 | 0.23 |
| C8H12OH+ | 125.0961 | 0.02 | 0.32 |
| C7H12N2H+ | 125.1073 | <10 ppt | 0.09 |
| C9H16H+ | 125.1325 | 0.04 | 0.49 |
| C5H4NO3+ | 126.0186 | <10 ppt | 0.17 |
| C6H8NS+ | 126.0372 | <10 ppt | 0.04 |
| C6H7NO2H+ | 126.055 | <10 ppt | 0.81 |
| C7H11NOH+ | 126.0913 | <10 ppt | 0.22 |
| C8H15NH+ | 126.1277 | <10 ppt | 0.05 |
| C5H2O4H+ | 127.0026 | 0.02 | 34.70 |
| C3F2H5O3+ | 127.0201 | <10 ppt | 0.10 |
| C6H6O3H+ | 127.039 | 0.01 | 1.18 |
| C10H7+ | 127.0542 | <10 ppt | 0.36 |
| C7H10O2H+ | 127.0754 | 0.02 | 0.37 |
| C8H14OH+ | 127.1117 | 0.07 | 9.00 |
| C5H5NO3H+ | 128.0342 | <10 ppt | 0.05 |
| C6H9NO2H+ | 128.0706 | <10 ppt | 0.13 |
| C7H13NOH+ | 128.107 | <10 ppt | 0.06 |
| C8H17NH+ | 128.1434 | <10 ppt | 0.03 |
| C6ClH5OH+ | 129.0102 | 0.01 | 10.23 |
| C5H4O4H+ | 129.0182 | <10 ppt | 0.13 |
| C4H5F4+ | 129.0322 | <10 ppt | 0.09 |
| C6H8O3H+ | 129.0546 | 0.02 | 0.27 |
| C10H8H+ | 129.0699 | 0.01 | 0.25 |
| C7H12O2H+ | 129.091 | 0.02 | 1.94 |
| C8H16OH+ | 129.1274 | 0.14 | 88.60 |
| C9H20H+ | 129.1638 | 0.01 | 0.38 |
| C4H3NO4H+ | 130.0135 | <10 ppt | 0.13 |
| C5H7NO3H+ | 130.0499 | <10 ppt | 0.37 |
| C9H8N+ | 130.0651 | <10 ppt | 0.10 |
| C6H11NO2H+ | 130.0863 | <10 ppt | 0.06 |
| C7H15NOH+ | 130.1226 | <10 ppt | 0.12 |
| C8H19NH+ | 130.159 | 0.01 | 0.14 |
| C2HCl3H+ | 130.9217 | <10 ppt | 0.16 |
| C4H2O5H+ | 130.9975 | 0.01 | 8.76 |
| C5H6O4H+ | 131.0339 | <10 ppt | 0.99 |
| C9H6OH+ | 131.0492 | <10 ppt | 0.08 |
| C6H10O3H+ | 131.0703 | 0.01 | 0.85 |
| C10H10H+ | 131.0855 | 0.01 | 2.19 |
| C7H14O2H+ | 131.1067 | 0.01 | 0.22 |
| C8H18OH+ | 131.143 | <10 ppt | 0.17 |
| C3HO5NH+ | 131.9928 | <10 ppt | 0.15 |
| C4H5NO4H+ | 132.0291 | <10 ppt | 0.08 |
| C8H5ONH+ | 132.0444 | <10 ppt | 0.02 |
| C5H9NO3H+ | 132.0655 | <10 ppt | 0.04 |
| C9H9NH+ | 132.0808 | <10 ppt | 0.27 |
| C6H13NO2H+ | 132.1019 | <10 ppt | 0.04 |
| C7H17NOH+ | 132.1383 | <10 ppt | 0.01 |
| C4H4O5H+ | 133.0132 | <10 ppt | 0.22 |
| C3H4N2O4H+ | 133.0244 | <10 ppt | 0.06 |
| C8H4O2H+ | 133.0284 | <10 ppt | 0.06 |
| C5H8O4H+ | 133.0495 | <10 ppt | 0.06 |
| C9H8OH+ | 133.0648 | <10 ppt | 0.10 |
| C8H8N2H+ | 133.076 | <10 ppt | 0.04 |
| C6H12O3H+ | 133.0859 | <10 ppt | 0.10 |
| C10H12H+ | 133.1012 | 0.02 | 0.29 |
| C7H16O2H+ | 133.1223 | 0.01 | 7.15 |
| C3H3NO5H+ | 134.0084 | <10 ppt | 0.01 |
| C7H3NO2H+ | 134.0237 | <10 ppt | 0.01 |
| C4H7NO4H+ | 134.0448 | <10 ppt | 0.02 |
| C8H7NOH+ | 134.06 | <10 ppt | 0.03 |
| C5H11NO3H+ | 134.0812 | <10 ppt | 0.02 |
| C9H11NH+ | 134.0964 | <10 ppt | 0.06 |
| C6H15NO2H+ | 134.1176 | <10 ppt | 0.01 |
| C7H19NOH+ | 134.154 | <10 ppt | 0.00 |
| C2ClF4+ | 134.9619 | <10 ppt | 0.02 |
| C4ClH3O3H+ | 134.9843 | <10 ppt | 0.18 |
| C4H6O3SH+ | 135.0111 | <10 ppt | 0.04 |
| C4H6O5H+ | 135.0288 | <10 ppt | 0.20 |
| C8H6O2H+ | 135.0441 | 0.01 | 0.82 |
| C5H10O4H+ | 135.0652 | 0.00 | 0.06 |
| C9H10OH+ | 135.0804 | 0.01 | 0.09 |
| C6H14O3H+ | 135.1016 | 0.01 | 0.33 |
| C10H14H+ | 135.1168 | 0.05 | 1.08 |
| C7H18O2H+ | 135.138 | <10 ppt | 0.31 |
| C7H5NSH+ | 136.0216 | 0.03 | 0.62 |
| C7H5O2NH+ | 136.0393 | <10 ppt | 0.03 |
| C4H9NO4H+ | 136.0604 | <10 ppt | 0.04 |
| C8H9NOH+ | 136.0757 | <10 ppt | 0.12 |
| C5H13NO3H+ | 136.0968 | <10 ppt | 0.04 |
| C9H13NH+ | 136.1121 | <10 ppt | 0.13 |
| C6H17NO2H+ | 136.1332 | <10 ppt | 0.04 |
| CFCl3H+ | 136.9122 | <10 ppt | 0.01 |
| C7H4OSH+ | 137.0056 | <10 ppt | 0.81 |
| C7H4O3H+ | 137.0233 | <10 ppt | 0.41 |
| C4H8O5H+ | 137.0445 | <10 ppt | 0.06 |
| C8H8O2H+ | 137.0597 | 0.06 | 0.18 |
| C5H12O4H+ | 137.0808 | <10 ppt | 0.07 |
| C9H12OH+ | 137.0961 | 0.01 | 0.18 |
| C8H12N2H+ | 137.1073 | <10 ppt | 0.10 |
| C6H16O3H+ | 137.1172 | 0.01 | 0.72 |
| C10H16H+ | 137.1325 | 0.29 | 35.21 |
| C6H3O3NH+ | 138.0186 | <10 ppt | 0.03 |
| C7H7NO2H+ | 138.055 | 0.02 | 0.24 |
| C4H11NO4H+ | 138.0761 | 0.04 | 0.11 |
| C8H11NOH+ | 138.0913 | <10 ppt | 0.03 |
| C9H15NH+ | 138.1277 | <10 ppt | 0.09 |
| C6H2O4H+ | 139.0026 | <10 ppt | 0.93 |
| C7H6OSH+ | 139.0212 | 0.01 | 0.20 |
| C7H6O3H+ | 139.039 | 0.01 | 0.25 |
| C4H10O3SH+ | 139.0423 | <10 ppt | 0.21 |
| C8H10SH+ | 139.0576 | 0.04 | 1.13 |
| C8H10O2H+ | 139.0754 | 0.69 | 3.96 |
| C9H14OH+ | 139.1117 | 0.07 | 0.29 |
| C10H18H+ | 139.1481 | 0.02 | 0.41 |
| C6H5NO3H+ | 140.0342 | 0.01 | 0.06 |
| C10H5NH+ | 140.0495 | 0.01 | 0.07 |
| C7H9NO2H+ | 140.0706 | <10 ppt | 1.40 |
| C6H9N3OH+ | 140.0818 | 0.01 | 0.11 |
| C8H13NOH+ | 140.107 | <10 ppt | 0.07 |
| C9H17NH+ | 140.1434 | <10 ppt | 0.05 |
| C6H4O4H+ | 141.0182 | 0.01 | 0.20 |
| C7H8OSH+ | 141.0369 | <10 ppt | 0.26 |
| C7H8O3H+ | 141.0546 | 0.01 | 0.12 |
| C11H8H+ | 141.0699 | <10 ppt | 0.09 |
| C8H12O2H+ | 141.091 | 0.01 | 0.13 |
| C9H16OH+ | 141.1274 | 0.02 | 0.19 |
| C10H20H+ | 141.1638 | 0.01 | 0.09 |
| C5H3NO4H+ | 142.0135 | <10 ppt | 0.02 |
| C6H8NOS+ | 142.0321 | <10 ppt | 0.02 |
| C6H7NO3H+ | 142.0499 | <10 ppt | 0.03 |
| C7H11NO2H+ | 142.0863 | <10 ppt | 0.08 |
| C8H15NOH+ | 142.1226 | <10 ppt | 0.02 |
| C9H19NH+ | 142.159 | <10 ppt | 0.01 |
| C5H2O5H+ | 142.9975 | <10 ppt | 0.50 |
| C6H6O2SH+ | 143.0161 | <10 ppt | 0.48 |
| C6H6O4H+ | 143.0339 | 0.00 | 0.21 |
| C7H10O3H+ | 143.0703 | 0.01 | 0.08 |
| C11H10H+ | 143.0855 | <10 ppt | 0.11 |
| C8H14O2H+ | 143.1067 | 0.02 | 0.34 |
| C9H18OH+ | 143.143 | 0.05 | 0.50 |
| C10H22H+ | 143.1794 | <10 ppt | 0.03 |
| C5H5NO4H+ | 144.0291 | <10 ppt | 0.02 |
| C9H5ONH+ | 144.0444 | <10 ppt | 0.02 |
| C6H9NO3H+ | 144.0655 | <10 ppt | 0.05 |
| C10H9NH+ | 144.0808 | <10 ppt | 0.04 |
| C7H13NO2H+ | 144.1019 | <10 ppt | 0.01 |
| C8H17NOH+ | 144.1383 | <10 ppt | 0.02 |
| C9H21NH+ | 144.1747 | <10 ppt | 0.01 |
| C6H2Cl2H+ | 144.9606 | <10 ppt | 9.78 |
| C4O6H+ | 144.9768 | <10 ppt | 8.58 |
| C5H5O3S+ | 144.9954 | <10 ppt | 0.03 |
| C5H4O5H+ | 145.0132 | <10 ppt | 0.07 |
| C6H9O2S+ | 145.0318 | <10 ppt | 0.09 |
| C6H8O4H+ | 145.0495 | <10 ppt | 0.07 |
| C10H8OH+ | 145.0648 | <10 ppt | 0.09 |
| C7H12O3H+ | 145.0859 | <10 ppt | 0.04 |
| C11H12H+ | 145.1012 | 0.01 | 0.16 |
| C8H16O2H+ | 145.1223 | 0.01 | 2.09 |
| C9H20OH+ | 145.1587 | <10 ppt | 0.09 |
| C8H4NO2+ | 146.0237 | <10 ppt | 0.12 |
| C5H7NO4H+ | 146.0448 | <10 ppt | 0.02 |
| C9H7ONH+ | 146.06 | <10 ppt | 0.03 |
| C6H11NO3H+ | 146.0812 | <10 ppt | 0.02 |
| C10H11NH+ | 146.0964 | <10 ppt | 0.03 |
| C7H15NO2H+ | 146.1176 | <10 ppt | 0.01 |
| C8H19NOH+ | 146.154 | <10 ppt | 0.00 |
| C5H6O5H+ | 147.0288 | <10 ppt | 75.36 |
| C9H6O2H+ | 147.0441 | 0.01 | 0.19 |
| C6H10O4H+ | 147.0652 | <10 ppt | 0.23 |
| C10H10OH+ | 147.0804 | <10 ppt | 0.32 |
| C7H14O3H+ | 147.1016 | <10 ppt | 0.37 |
| C11H14H+ | 147.1168 | 0.01 | 0.35 |
| C8H18O2H+ | 147.138 | 0.01 | 1.03 |
| C8H5NO2H+ | 148.0393 | <10 ppt | 0.01 |
| C5H9O4NH+ | 148.0604 | <10 ppt | 0.02 |
| C9H9NOH+ | 148.0757 | <10 ppt | 0.03 |
| C6H13NO3H+ | 148.0968 | <10 ppt | 0.03 |
| C10H13NH+ | 148.1121 | <10 ppt | 0.03 |
| C7H17NO2H+ | 148.1332 | <10 ppt | 0.01 |
| C8H21NOH+ | 148.1696 | <10 ppt | 0.02 |
| C3H2F5O+ | 149.002 | 1.00 | 1002.03 |
| C8H4O3H+ | 149.0233 | 0.05 | 776.40 |
| C4H5F5H+ | 149.0384 | 0.01 | 0.25 |
| C5H8O5H+ | 149.0445 | 0.01 | 3.89 |
| C9H8O2H+ | 149.0597 | 0.02 | 0.34 |
| C6H12O4H+ | 149.0808 | 0.01 | 0.38 |
| C10H12OH+ | 149.0961 | 0.01 | 0.43 |
| C7H16O3H+ | 149.1172 | <10 ppt | 0.44 |
| C11H16H+ | 149.1325 | 0.02 | 1.61 |
| C8H20O2H+ | 149.1536 | <10 ppt | 0.03 |
| C3H4NO6+ | 150.0033 | 0.01 | 11.42 |
| C7H3NO3H+ | 150.0186 | <10 ppt | 1.05 |
| C4H7NO5H+ | 150.0397 | <10 ppt | 0.08 |
| C8H7NO2H+ | 150.055 | <10 ppt | 0.06 |
| C5H11NO4H+ | 150.0761 | <10 ppt | 0.06 |
| C9H11NOH+ | 150.0913 | <10 ppt | 0.04 |
| C6H15NO3H+ | 150.1125 | <10 ppt | 0.03 |
| C10H15NH+ | 150.1277 | <10 ppt | 0.07 |
| C7H19NO2H+ | 150.1489 | <10 ppt | 0.02 |
| C3F6H+ | 150.9977 | <10 ppt | 0.35 |
| C4H6O6H+ | 151.0237 | <10 ppt | 0.16 |
| C8H6O3H+ | 151.039 | <10 ppt | 0.07 |
| C5H10O5H+ | 151.0601 | <10 ppt | 0.04 |
| C9H10O2H+ | 151.0754 | <10 ppt | 0.07 |
| C6H14O4H+ | 151.0965 | <10 ppt | 0.15 |
| C10H14OH+ | 151.1117 | 0.02 | 3.89 |
| C7H18O3H+ | 151.1329 | <10 ppt | 0.07 |
| C11H18H+ | 151.1481 | 0.01 | 0.24 |
| C7H5NOSH+ | 152.0165 | <10 ppt | 0.02 |
| C7H5NO3H+ | 152.0342 | <10 ppt | 0.01 |
| C8H9NO2H+ | 152.0706 | <10 ppt | 0.09 |
| C5H13NO4H+ | 152.0917 | <10 ppt | 0.02 |
| C9H13NOH+ | 152.107 | <10 ppt | 0.02 |
| C10H17NH+ | 152.1434 | <10 ppt | 0.06 |
| CCl4H+ | 152.8827 | <10 ppt | 0.04 |
| C7H4O4H+ | 153.0182 | <10 ppt | 0.12 |
| C4H8O6H+ | 153.0394 | <10 ppt | 0.06 |
| C8H8O3H+ | 153.0546 | 0.02 | 0.17 |
| C12H8H+ | 153.0699 | <10 ppt | 0.10 |
| C9H12SH+ | 153.0732 | <10 ppt | 0.05 |
| C9H12O2H+ | 153.091 | 0.01 | 0.11 |
| C10H16OH+ | 153.1274 | 0.02 | 0.58 |
| C11H20H+ | 153.1638 | 0.01 | 0.07 |
| C2H4NO7+ | 153.9982 | <10 ppt | 0.01 |
| C6H3O4NH+ | 154.0135 | <10 ppt | 0.01 |
| C7H7NO3H+ | 154.0499 | <10 ppt | 0.02 |
| C11H7NH+ | 154.0651 | <10 ppt | 0.03 |
| C8H11NO2H+ | 154.0863 | <10 ppt | 2.31 |
| C9H15NOH+ | 154.1226 | <10 ppt | 0.08 |
| C10H19NH+ | 154.159 | <10 ppt | 0.03 |
| C2ClF5H+ | 154.9681 | <10 ppt | 0.57 |
| C5H2N2O4H+ | 155.0087 | <10 ppt | 0.65 |
| C7H6O4H+ | 155.0339 | <10 ppt | 0.08 |
| C6H6N2O3H+ | 155.0451 | <10 ppt | 0.14 |
| C8H10O3H+ | 155.0703 | 0.01 | 0.06 |
| C12H10H+ | 155.0855 | <10 ppt | 0.07 |
| C9H14SH+ | 155.0889 | <10 ppt | 0.09 |
| C9H15O2+ | 155.1067 | 0.01 | 0.13 |
| C6H19O4+ | 155.1278 | <10 ppt | 0.17 |
| C10H18OH+ | 155.143 | 0.02 | 0.82 |
| C11H22H+ | 155.1794 | 0.01 | 0.06 |
| C6H5NO4H+ | 156.0291 | <10 ppt | 0.06 |
| C5H5N3O3H+ | 156.0404 | <10 ppt | 0.02 |
| C7H9NO3H+ | 156.0655 | <10 ppt | 0.02 |
| C11H9NH+ | 156.0808 | <10 ppt | 0.02 |
| C8H13NO2H+ | 156.1019 | <10 ppt | 0.05 |
| C9H17NOH+ | 156.1383 | <10 ppt | 0.08 |
| C10H21NH+ | 156.1747 | <10 ppt | 0.01 |
| C6H4O5H+ | 157.0132 | <10 ppt | 0.26 |
| C10H4O2H+ | 157.0284 | <10 ppt | 0.11 |
| C7H8O4H+ | 157.0495 | <10 ppt | 0.08 |
| C11H8OH+ | 157.0648 | <10 ppt | 0.08 |
| C8H12O3H+ | 157.0859 | <10 ppt | 0.07 |
| C12H12H+ | 157.1012 | <10 ppt | 0.07 |
| C9H16O2H+ | 157.1223 | 0.01 | 0.12 |
| C10H20OH+ | 157.1587 | 0.03 | 0.90 |
| C11H24H+ | 157.1951 | <10 ppt | 0.02 |
| C5H3NO5H+ | 158.0084 | <10 ppt | 0.02 |
| C6H7NO4H+ | 158.0448 | <10 ppt | 0.02 |
| C10H7ONH+ | 158.06 | <10 ppt | 0.01 |
| C7H11NO3H+ | 158.0812 | <10 ppt | 0.07 |
| C11H11NH+ | 158.0964 | <10 ppt | 0.03 |
| C8H15NO2H+ | 158.1176 | <10 ppt | 0.03 |
| C9H19NOH+ | 158.154 | 0.03 | 0.58 |
| C10H23NH+ | 158.1903 | <10 ppt | 0.05 |
| C5H2O4SH+ | 158.9747 | <10 ppt | 0.01 |
| C5H2O6H+ | 158.9924 | <10 ppt | 0.02 |
| C6H6SO3H+ | 159.0111 | <10 ppt | 0.04 |
| C6H6O5H+ | 159.0288 | <10 ppt | 0.09 |
| C10H6O2H+ | 159.0441 | <10 ppt | 0.27 |
| C7H10O4H+ | 159.0652 | <10 ppt | 0.60 |
| C11H10OH+ | 159.0804 | <10 ppt | 0.07 |
| C8H14O3H+ | 159.1016 | <10 ppt | 0.06 |
| C12H14H+ | 159.1168 | 0.01 | 0.10 |
| C9H18O2H+ | 159.138 | 0.01 | 0.08 |
| C10H22OH+ | 159.1744 | <10 ppt | 0.02 |
| C5H5NO5H+ | 160.0241 | <10 ppt | 0.01 |
| C9H5NO2H+ | 160.0393 | <10 ppt | 0.01 |
| C6H9NO4H+ | 160.0604 | <10 ppt | 0.01 |
| C10H9ONH+ | 160.0757 | <10 ppt | 0.01 |
| C9H9N3H+ | 160.0869 | <10 ppt | 0.01 |
| C7H13NO3H+ | 160.0968 | <10 ppt | 0.01 |
| C11H13NH+ | 160.1121 | <10 ppt | 0.01 |
| C8H17NO2H+ | 160.1332 | <10 ppt | 0.01 |
| C9H21NOH+ | 160.1696 | <10 ppt | 0.00 |
| C3F4O3H+ | 160.9856 | <10 ppt | 0.41 |
| C6H5O3ClH+ | 161 | <10 ppt | 0.39 |
| C6H8OS2H+ | 161.0089 | <10 ppt | 0.14 |
| C10H8SH+ | 161.042 | <10 ppt | 0.05 |
| C6H8O5H+ | 161.0445 | <10 ppt | 0.06 |
| C10H8O2H+ | 161.0597 | <10 ppt | 0.05 |
| C7H12O4H+ | 161.0808 | <10 ppt | 0.04 |
| C11H12OH+ | 161.0961 | 0.01 | 0.09 |
| C8H16O3H+ | 161.1172 | <10 ppt | 0.07 |
| C12H16H+ | 161.1325 | <10 ppt | 0.17 |
| C9H20SH+ | 161.1359 | <10 ppt | 0.18 |
| C9H20O2H+ | 161.1536 | 0.01 | 0.15 |
| C5H7NO5H+ | 162.0397 | <10 ppt | 0.01 |
| C9H7NO2H+ | 162.055 | <10 ppt | 0.01 |
| C6H11NO4H+ | 162.0761 | <10 ppt | 0.01 |
| C10H11NOH+ | 162.0913 | <10 ppt | 0.01 |
| C7H15NO3H+ | 162.1125 | <10 ppt | 0.01 |
| C11H15NH+ | 162.1277 | <10 ppt | 0.02 |
| C8H19NO2H+ | 162.1489 | <10 ppt | 0.01 |
| C9H23NOH+ | 162.1852 | <10 ppt | 0.01 |
| C5H7O4S+ | 163.006 | <10 ppt | 0.03 |
| C6H11OS2+ | 163.0246 | <10 ppt | 0.05 |
| C9H6O3H+ | 163.039 | <10 ppt | 0.05 |
| C6H10O5H+ | 163.0601 | <10 ppt | 0.10 |
| C10H10O2H+ | 163.0754 | 0.01 | 0.27 |
| C7H14O4H+ | 163.0965 | 0.01 | 0.53 |
| C11H14OH+ | 163.1117 | 0.01 | 0.16 |
| C8H18O3H+ | 163.1329 | 0.01 | 0.14 |
| C12H18H+ | 163.1481 | 0.01 | 0.18 |
| C9H22O2H+ | 163.1693 | <10 ppt | 1.21 |
| C4H5NO6H+ | 164.019 | 0.01 | 0.20 |
| C8H5NO3H+ | 164.0342 | <10 ppt | 0.04 |
| C12H5NH+ | 164.0495 | <10 ppt | 0.03 |
| C5H9NO5H+ | 164.0553 | <10 ppt | 0.02 |
| C9H9O2NH+ | 164.0706 | <10 ppt | 0.02 |
| C6H13NO4H+ | 164.0917 | <10 ppt | 0.04 |
| C10H13NOH+ | 164.107 | <10 ppt | 0.02 |
| C7H17NO3H+ | 164.1281 | <10 ppt | 0.01 |
| C11H17NH+ | 164.1434 | <10 ppt | 0.01 |
| C8H21NO2H+ | 164.1645 | <10 ppt | 0.01 |
| C3H2ClF4O+ | 164.9725 | 0.02 | 81.60 |
| C8H4O4H+ | 165.0182 | 0.01 | 14.43 |
| C5H8O6H+ | 165.0394 | <10 ppt | 0.21 |
| C9H8O3H+ | 165.0546 | <10 ppt | 0.19 |
| C6H12O5H+ | 165.0757 | <10 ppt | 0.19 |
| C10H12O2H+ | 165.091 | 0.01 | 0.28 |
| C7H16O4H+ | 165.1121 | <10 ppt | 0.17 |
| C11H16OH+ | 165.1274 | <10 ppt | 0.06 |
| C8H20O3H+ | 165.1485 | <10 ppt | 0.05 |
| C12H20H+ | 165.1638 | 0.01 | 0.12 |
| C3H3NO7H+ | 165.9982 | <10 ppt | 1.41 |
| C7H3NO4H+ | 166.0135 | <10 ppt | 0.03 |
| C4H7NO6H+ | 166.0346 | <10 ppt | 0.04 |
| C8H7NO3H+ | 166.0499 | <10 ppt | 0.01 |
| C5H11NO5H+ | 166.071 | <10 ppt | 0.01 |
| C9H11NO2H+ | 166.0863 | <10 ppt | 0.04 |
| C6H15NO4H+ | 166.1074 | <10 ppt | 0.01 |
| C10H15NOH+ | 166.1226 | <10 ppt | 0.01 |
| C4H7O7+ | 167.0186 | <10 ppt | 2.29 |
| C8H6O4H+ | 167.0339 | <10 ppt | 0.06 |
| C12H6OH+ | 167.0492 | 0.01 | 0.43 |
| C9H10O3H+ | 167.0703 | 0.01 | 0.17 |
| C13H10H+ | 167.0855 | <10 ppt | 0.21 |
| C10H14SH+ | 167.0889 | <10 ppt | 0.07 |
| C10H14O2H+ | 167.1067 | 0.01 | 0.08 |
| C11H18OH+ | 167.143 | <10 ppt | 0.09 |
| C12H22H+ | 167.1794 | <10 ppt | 0.04 |
| C7H5NO4H+ | 168.0291 | <10 ppt | 0.01 |
| C11H5NOH+ | 168.0444 | <10 ppt | 0.01 |
| C8H9NO3H+ | 168.0655 | <10 ppt | 0.04 |
| C12H9NH+ | 168.0808 | <10 ppt | 0.04 |
| C9H13NO2H+ | 168.1019 | <10 ppt | 0.40 |
| C10H17NOH+ | 168.1383 | <10 ppt | 0.03 |
| C11H21NH+ | 168.1747 | <10 ppt | 0.02 |
| C5H3Cl3H+ | 168.9373 | <10 ppt | 0.01 |
| C3H2F6OH+ | 169.0083 | <10 ppt | 0.02 |
| C7H4O5H+ | 169.0132 | <10 ppt | 0.03 |
| C6H4N2O4H+ | 169.0244 | <10 ppt | 0.07 |
| C8H8O4H+ | 169.0495 | <10 ppt | 0.08 |
| C12H9O+ | 169.0648 | <10 ppt | 0.04 |
| C9H12O3H+ | 169.0859 | <10 ppt | 0.05 |
| C10H16SH+ | 169.1046 | <10 ppt | 0.07 |
| C10H16O2H+ | 169.1223 | <10 ppt | 0.12 |
| C11H20OH+ | 169.1587 | 0.01 | 0.11 |
| C12H24H+ | 169.1951 | <10 ppt | 0.05 |
| C6H3NO5H+ | 170.0084 | <10 ppt | 0.01 |
| C7H7NO4H+ | 170.0448 | <10 ppt | 0.01 |
| C11H7ONH+ | 170.06 | <10 ppt | 0.08 |
| C8H11NO3H+ | 170.0812 | <10 ppt | 0.11 |
| C12H11NH+ | 170.0964 | <10 ppt | 0.01 |
| C9H15NO2H+ | 170.1176 | <10 ppt | 0.01 |
| C10H19NOH+ | 170.154 | <10 ppt | 0.01 |
| C2Cl2F4H+ | 170.9386 | <10 ppt | 0.51 |
| C3HF7H+ | 171.0039 | <10 ppt | 0.03 |
| C7H6O5H+ | 171.0288 | <10 ppt | 0.04 |
| C8H10O4H+ | 171.0652 | <10 ppt | 0.43 |
| C12H11O+ | 171.0804 | 0.05 | 2.65 |
| C9H14O3H+ | 171.1016 | <10 ppt | 0.09 |
| C13H14H+ | 171.1168 | <10 ppt | 0.10 |
| C10H18O2H+ | 171.138 | 0.01 | 0.13 |
| C9H18N2OH+ | 171.1492 | <10 ppt | 0.08 |
| C11H22OH+ | 171.1744 | 0.03 | 0.19 |
| C12H26H+ | 171.2107 | <10 ppt | 0.02 |
| C6H5NO5H+ | 172.0241 | <10 ppt | 0.01 |
| C10H5O2NH+ | 172.0393 | <10 ppt | 0.02 |
| C7H9NO4H+ | 172.0604 | <10 ppt | 0.03 |
| C8H13NO3H+ | 172.0968 | <10 ppt | 0.13 |
| C9H17NO2H+ | 172.1332 | <10 ppt | 0.01 |
| C10H21NOH+ | 172.1696 | <10 ppt | 0.01 |
| C5H7Cl3H+ | 172.9686 | <10 ppt | 0.02 |
| C2H5O9+ | 172.9928 | <10 ppt | 0.08 |
| C6H4O6H+ | 173.0081 | <10 ppt | 0.06 |
| C10H4O3H+ | 173.0233 | <10 ppt | 0.29 |
| C7H8O5H+ | 173.0445 | <10 ppt | 0.05 |
| C11H8O2H+ | 173.0597 | <10 ppt | 0.46 |
| C8H12O4H+ | 173.0808 | <10 ppt | 0.04 |
| C12H12OH+ | 173.0961 | <10 ppt | 0.04 |
| C9H16O3H+ | 173.1172 | <10 ppt | 0.05 |
| C13H16H+ | 173.1325 | <10 ppt | 0.10 |
| C10H20O2H+ | 173.1536 | 0.01 | 0.09 |
| C5H3NO6H+ | 174.0033 | <10 ppt | 0.02 |
| C9H3NO3H+ | 174.0186 | <10 ppt | 0.03 |
| C6H7NO5H+ | 174.0397 | <10 ppt | 0.03 |
| C10H7NO2H+ | 174.055 | <10 ppt | 0.03 |
| C7H11NO4H+ | 174.0761 | <10 ppt | 0.02 |
| C11H11NOH+ | 174.0913 | <10 ppt | 0.01 |
| C8H15NO3H+ | 174.1125 | <10 ppt | 0.01 |
| C12H15NH+ | 174.1277 | <10 ppt | 0.03 |
| C9H19NO2H+ | 174.1489 | <10 ppt | 0.01 |
| C10H23NOH+ | 174.1852 | <10 ppt | 0.00 |
| C9H3O4+ | 175.0026 | <10 ppt | 0.53 |
| C6H6O6H+ | 175.0237 | <10 ppt | 0.06 |
| C10H6O3H+ | 175.039 | <10 ppt | 0.04 |
| C7H10O5H+ | 175.0601 | <10 ppt | 0.04 |
| C11H10O2H+ | 175.0754 | <10 ppt | 0.18 |
| C8H14O4H+ | 175.0965 | <10 ppt | 0.02 |
| C12H14OH+ | 175.1117 | <10 ppt | 0.03 |
| C9H18O3H+ | 175.1329 | 0.01 | 0.90 |
| C13H18H+ | 175.1481 | 0.18 | 4.10 |
| C10H22SH+ | 175.1515 | 0.01 | 2.08 |
| C10H22O2H+ | 175.1693 | 0.01 | 0.43 |
| C5H5NO6H+ | 176.019 | <10 ppt | 0.02 |
| C9H5O3NH+ | 176.0342 | <10 ppt | 0.01 |
| C6H9NO5H+ | 176.0553 | <10 ppt | 0.01 |
| C10H9O2NH+ | 176.0706 | <10 ppt | 0.01 |
| C7H13NO4H+ | 176.0917 | <10 ppt | 0.01 |
| C11H13NOH+ | 176.107 | <10 ppt | 0.00 |
| C8H17NO3H+ | 176.1281 | <10 ppt | 0.03 |
| C12H17NH+ | 176.1434 | <10 ppt | 0.15 |
| C9H21NO2H+ | 176.1645 | <10 ppt | 0.03 |
| C10H25NOH+ | 176.2009 | <10 ppt | 0.02 |
| C5H4O7H+ | 177.003 | <10 ppt | 0.18 |
| C9H4O4H+ | 177.0182 | <10 ppt | 0.03 |
| C6H8O6H+ | 177.0394 | <10 ppt | 0.04 |
| C10H8O3H+ | 177.0546 | <10 ppt | 0.12 |
| C7H12O5H+ | 177.0757 | <10 ppt | 0.02 |
| C11H13O2+ | 177.091 | <10 ppt | 0.03 |
| C8H16O4H+ | 177.1121 | <10 ppt | 0.05 |
| C12H16OH+ | 177.1274 | <10 ppt | 0.07 |
| C9H20O3H+ | 177.1485 | <10 ppt | 0.54 |
| C13H20H+ | 177.1638 | 0.02 | 1.76 |
| C10H24O2H+ | 177.1849 | <10 ppt | 0.05 |
| C9H7NOSH+ | 178.0321 | <10 ppt | 0.01 |
| C9H7NO3H+ | 178.0499 | <10 ppt | 0.01 |
| C6H11NO5H+ | 178.071 | <10 ppt | 0.01 |
| C7H15NO4H+ | 178.1074 | <10 ppt | 0.01 |
| C11H15NOH+ | 178.1226 | <10 ppt | 0.02 |
| C8H19NO3H+ | 178.1438 | <10 ppt | 0.04 |
| C12H19NH+ | 178.159 | <10 ppt | 0.05 |
| C9H23NO2H+ | 178.1802 | <10 ppt | 0.01 |
| C6Cl3HH+ | 178.9217 | <10 ppt | 0.05 |
| C3H3N2O7+ | 178.9935 | <10 ppt | 0.06 |
| C9H6O2SH+ | 179.0161 | <10 ppt | 0.10 |
| C9H6O4H+ | 179.0339 | <10 ppt | 0.02 |
| C6H11O6+ | 179.055 | <10 ppt | 0.03 |
| C10H10O3H+ | 179.0703 | <10 ppt | 0.04 |
| C14H10H+ | 179.0855 | <10 ppt | 0.03 |
| C7H14O5H+ | 179.0914 | <10 ppt | 0.03 |
| C11H14O2H+ | 179.1067 | <10 ppt | 0.03 |
| C8H18O4H+ | 179.1278 | <10 ppt | 0.09 |
| C12H18OH+ | 179.143 | <10 ppt | 0.37 |
| C9H22O3H+ | 179.1642 | <10 ppt | 0.04 |
| C13H22H+ | 179.1794 | <10 ppt | 0.06 |
| C4H6NO7+ | 180.0139 | <10 ppt | 0.16 |
| C9H10NO3+ | 180.0655 | <10 ppt | 0.07 |
| C10H13NO2H+ | 180.1019 | <10 ppt | 0.03 |
| C7H17NO4H+ | 180.123 | <10 ppt | 0.03 |
| C11H17NOH+ | 180.1383 | <10 ppt | 0.02 |
| C12H21NH+ | 180.1747 | <10 ppt | 0.18 |
| C4H5O8+ | 180.9979 | 0.07 | 678.64 |
| C4H3F6O+ | 181.0083 | 4.01 | 757.78 |
| C9H8SO2H+ | 181.0318 | 0.44 | 732.15 |
| C9H8O4H+ | 181.0495 | 0.01 | 644.00 |
| C10H12O3H+ | 181.0859 | 0.03 | 383.85 |
| C11H16SH+ | 181.1046 | 0.01 | 166.78 |
| C10H17N2O+ | 181.1335 | 0.01 | 211.50 |
| C12H20OH+ | 181.1587 | <10 ppt | 161.86 |
| C13H24H+ | 181.1951 | <10 ppt | 173.13 |
| C4H7NO7H+ | 182.0295 | 0.01 | 33.39 |
| C8H7NO4H+ | 182.0448 | <10 ppt | 0.05 |
| C12H7NOH+ | 182.06 | <10 ppt | 0.07 |
| C9H11NO3H+ | 182.0812 | <10 ppt | 0.12 |
| C13H12N+ | 182.0964 | <10 ppt | 0.13 |
| C10H15NO2H+ | 182.1176 | <10 ppt | 0.14 |
| C11H19NOH+ | 182.154 | <10 ppt | 0.04 |
| C12H23NH+ | 182.1903 | <10 ppt | 1.38 |
| C8H6O5H+ | 183.0288 | <10 ppt | 8.44 |
| C9H11O4+ | 183.0652 | <10 ppt | 0.10 |
| C10H15OS+ | 183.0838 | <10 ppt | 0.14 |
| C10H14O3H+ | 183.1016 | <10 ppt | 0.04 |
| C14H14H+ | 183.1168 | <10 ppt | 0.03 |
| C11H18O2H+ | 183.138 | <10 ppt | 0.07 |
| C12H22OH+ | 183.1744 | <10 ppt | 0.10 |
| C13H26H+ | 183.2107 | <10 ppt | 0.10 |
| C7H5NO5H+ | 184.0241 | <10 ppt | 0.29 |
| C11H5NO2H+ | 184.0393 | <10 ppt | 0.02 |
| C8H9NO4H+ | 184.0604 | <10 ppt | 0.01 |
| C12H9NOH+ | 184.0757 | <10 ppt | 0.01 |
| C9H13NO3H+ | 184.0968 | <10 ppt | 0.02 |
| C13H13NH+ | 184.1121 | <10 ppt | 0.02 |
| C10H17NO2H+ | 184.1332 | <10 ppt | 0.01 |
| C11H22NO+ | 184.1696 | <10 ppt | 0.03 |
| C3H2ClF5OH+ | 184.9787 | <10 ppt | 0.07 |
| C7H4O6H+ | 185.0081 | <10 ppt | 0.03 |
| C4H8O8H+ | 185.0292 | <10 ppt | 0.04 |
| C8H8O5H+ | 185.0445 | <10 ppt | 0.05 |
| C12H8O2H+ | 185.0597 | <10 ppt | 0.21 |
| C9H12O4H+ | 185.0808 | <10 ppt | 0.03 |
| C13H12OH+ | 185.0961 | <10 ppt | 0.03 |
| C10H16O3H+ | 185.1172 | <10 ppt | 0.04 |
| C14H16H+ | 185.1325 | <10 ppt | 0.04 |
| C11H20O2H+ | 185.1536 | 0.01 | 0.09 |
| C12H24OH+ | 185.19 | 0.05 | 0.26 |
| C13H28H+ | 185.2264 | <10 ppt | 0.09 |
| C7H7NO5H+ | 186.0397 | <10 ppt | 0.02 |
| C8H11NO4H+ | 186.0761 | <10 ppt | 0.02 |
| C12H11NOH+ | 186.0913 | <10 ppt | 0.01 |
| C9H15NO3H+ | 186.1125 | <10 ppt | 0.05 |
| C13H15NH+ | 186.1277 | <10 ppt | 0.01 |
| C10H19NO2H+ | 186.1489 | <10 ppt | 0.01 |
| C11H23NOH+ | 186.1852 | <10 ppt | 0.02 |
| C7H6O6H+ | 187.0237 | <10 ppt | 0.09 |
| C11H6O3H+ | 187.039 | <10 ppt | 0.03 |
| C8H10O5H+ | 187.0601 | <10 ppt | 0.02 |
| C12H10O2H+ | 187.0754 | <10 ppt | 0.03 |
| C9H14O4H+ | 187.0965 | <10 ppt | 0.02 |
| C13H14OH+ | 187.1117 | <10 ppt | 0.02 |
| C10H18O3H+ | 187.1329 | <10 ppt | 0.13 |
| C14H18H+ | 187.1481 | <10 ppt | 0.42 |
| C11H22O2H+ | 187.1693 | <10 ppt | 0.04 |
| C12H26OH+ | 187.2056 | <10 ppt | 0.02 |
| C7H9NO5H+ | 188.0553 | <10 ppt | 0.01 |
| C11H9NO2H+ | 188.0706 | <10 ppt | 0.04 |
| C8H13NO4H+ | 188.0917 | <10 ppt | 0.01 |
| C12H13NOH+ | 188.107 | <10 ppt | 0.01 |
| C9H17NO3H+ | 188.1281 | <10 ppt | 0.01 |
| C13H17NH+ | 188.1434 | <10 ppt | 0.02 |
| C10H21NO2H+ | 188.1645 | <10 ppt | 0.01 |
| C11H25NOH+ | 188.2009 | <10 ppt | 0.01 |
| C6H4O7H+ | 189.003 | <10 ppt | 0.02 |
| C10H5O4+ | 189.0182 | <10 ppt | 0.05 |
| C7H8O6H+ | 189.0394 | <10 ppt | 0.03 |
| C11H8O3H+ | 189.0546 | <10 ppt | 0.02 |
| C8H12O5H+ | 189.0757 | <10 ppt | 0.02 |
| C12H12O2H+ | 189.091 | <10 ppt | 0.02 |
| C9H16O4H+ | 189.1121 | <10 ppt | 0.93 |
| C13H16OH+ | 189.1274 | <10 ppt | 4.56 |
| C10H20O3H+ | 189.1485 | <10 ppt | 0.13 |
| C14H20H+ | 189.1638 | 0.01 | 0.15 |
| C11H24O2H+ | 189.1849 | <10 ppt | 0.16 |
| C10H7NO3H+ | 190.0499 | <10 ppt | 0.01 |
| C11H12NO2+ | 190.0863 | <10 ppt | 0.01 |
| C12H15NOH+ | 190.1226 | <10 ppt | 0.04 |
| C13H19NH+ | 190.159 | 0.01 | 0.11 |
| C9H3O5+ | 190.9975 | <10 ppt | 0.07 |
| C10H6O4H+ | 191.0339 | <10 ppt | 0.03 |
| C7H10O6H+ | 191.055 | <10 ppt | 0.02 |
| C11H10O3H+ | 191.0703 | <10 ppt | 0.01 |
| C15H10H+ | 191.0855 | <10 ppt | 0.01 |
| C8H14O5H+ | 191.0914 | <10 ppt | 0.01 |
| C12H14O2H+ | 191.1067 | <10 ppt | 0.02 |
| C9H18O4H+ | 191.1278 | <10 ppt | 0.04 |
| C13H18OH+ | 191.143 | <10 ppt | 0.09 |
| C10H22O3H+ | 191.1642 | <10 ppt | 0.32 |
| C14H22H+ | 191.1794 | 0.01 | 0.76 |
| C6H10NO4S+ | 192.0325 | <10 ppt | 0.04 |
| C6H10NO6+ | 192.0503 | <10 ppt | 0.05 |
| C10H10NO3+ | 192.0655 | <10 ppt | 0.03 |
| C7H14NO5+ | 192.0867 | <10 ppt | 0.01 |
| C11H14NO2+ | 192.1019 | <10 ppt | 0.01 |
| C8H18NO4+ | 192.123 | <10 ppt | 0.02 |
| C12H18NO+ | 192.1383 | <10 ppt | 0.02 |
| C13H21NH+ | 192.1747 | <10 ppt | 0.02 |
| C6H8O7H+ | 193.0343 | <10 ppt | 0.21 |
| C10H8O4H+ | 193.0495 | <10 ppt | 0.04 |
| C14H8OH+ | 193.0648 | <10 ppt | 0.04 |
| C7H12O6H+ | 193.0707 | <10 ppt | 0.03 |
| C11H12O3H+ | 193.0859 | <10 ppt | 0.04 |
| C15H12H+ | 193.1012 | <10 ppt | 0.02 |
| C8H16O5H+ | 193.1071 | <10 ppt | 0.02 |
| C12H16O2H+ | 193.1223 | <10 ppt | 0.14 |
| C13H20OH+ | 193.1587 | 0.01 | 0.65 |
| C14H24H+ | 193.1951 | 0.01 | 0.16 |
| C9H7NO4H+ | 194.0448 | <10 ppt | 0.02 |
| C10H11NO3H+ | 194.0812 | <10 ppt | 0.01 |
| C7H15NO5H+ | 194.1023 | <10 ppt | 0.01 |
| C11H15NO2H+ | 194.1176 | <10 ppt | 0.01 |
| C12H19NOH+ | 194.154 | <10 ppt | 0.02 |
| C13H23NH+ | 194.1903 | <10 ppt | 0.01 |
| C8H2O6H+ | 194.9924 | <10 ppt | 0.13 |
| C4F5H4O3+ | 195.0075 | <10 ppt | 0.07 |
| C9H6O5H+ | 195.0288 | <10 ppt | 0.03 |
| C13H6O2H+ | 195.0441 | <10 ppt | 0.02 |
| C10H10O4H+ | 195.0652 | <10 ppt | 0.02 |
| C14H10OH+ | 195.0804 | <10 ppt | 0.05 |
| C11H14O3H+ | 195.1016 | <10 ppt | 0.02 |
| C15H14H+ | 195.1168 | <10 ppt | 0.03 |
| C12H18O2H+ | 195.138 | <10 ppt | 0.14 |
| C13H22OH+ | 195.1744 | 0.01 | 1.95 |
| C14H26H+ | 195.2107 | <10 ppt | 0.03 |
| C2HBrClF3+ | 195.8897 | <10 ppt | 0.01 |
| C9H9NO4H+ | 196.0604 | <10 ppt | 0.01 |
| C10H13NO3H+ | 196.0968 | <10 ppt | 0.01 |
| C14H13NH+ | 196.1121 | <10 ppt | 0.02 |
| C11H17NO2H+ | 196.1332 | <10 ppt | 0.20 |
| C12H21NOH+ | 196.1696 | <10 ppt | 0.02 |
| C13H25NH+ | 196.206 | <10 ppt | 0.01 |
| C6H3Cl3OH+ | 196.9322 | <10 ppt | 0.03 |
| C8H4O6H+ | 197.0081 | <10 ppt | 0.59 |
| C12H4O3H+ | 197.0233 | <10 ppt | 0.02 |
| C9H8O5H+ | 197.0445 | <10 ppt | 0.28 |
| C13H8O2H+ | 197.0597 | <10 ppt | 0.03 |
| C10H12O4H+ | 197.0808 | <10 ppt | 0.01 |
| C14H12OH+ | 197.0961 | <10 ppt | 0.03 |
| C11H16O3H+ | 197.1172 | <10 ppt | 0.02 |
| C15H16H+ | 197.1325 | <10 ppt | 0.03 |
| C12H20O2H+ | 197.1536 | 0.01 | 0.07 |
| C13H24OH+ | 197.19 | <10 ppt | 0.06 |
| C14H28H+ | 197.2264 | 0.01 | 0.25 |
| C8H7NO5H+ | 198.0397 | <10 ppt | 0.61 |
| C9H11NO4H+ | 198.0761 | <10 ppt | 0.01 |
| C10H15NO3H+ | 198.1125 | <10 ppt | 0.03 |
| C11H19NO2H+ | 198.1489 | <10 ppt | 0.01 |
| C12H23NOH+ | 198.1852 | <10 ppt | 0.02 |
| C13H27NH+ | 198.2216 | <10 ppt | 0.04 |
| C8H6O6H+ | 199.0237 | <10 ppt | 7.72 |
| C12H6O3H+ | 199.039 | <10 ppt | 0.25 |
| C9H10O5H+ | 199.0601 | <10 ppt | 0.20 |
| C13H10O2H+ | 199.0754 | <10 ppt | 0.20 |
| C10H14O4H+ | 199.0965 | <10 ppt | 0.26 |
| C14H14OH+ | 199.1117 | <10 ppt | 0.19 |
| C11H18O3H+ | 199.1329 | <10 ppt | 0.15 |
| C15H18H+ | 199.1481 | <10 ppt | 0.10 |
| C12H22O2H+ | 199.1693 | 0.01 | 0.07 |
| C13H26OH+ | 199.2056 | 0.01 | 0.08 |
| C14H30H+ | 199.242 | <10 ppt | 0.01 |
| C6H6N3O5+ | 200.0302 | <10 ppt | 0.04 |
| C8H9NO5H+ | 200.0553 | <10 ppt | 0.03 |
| C9H13NO4H+ | 200.0917 | <10 ppt | 0.03 |
| C10H17NO3H+ | 200.1281 | <10 ppt | 0.02 |
| C11H21NO2H+ | 200.1645 | <10 ppt | 0.01 |
| C12H25NOH+ | 200.2009 | <10 ppt | 0.01 |
| C13H29NH+ | 200.2373 | <10 ppt | 0.01 |
| C4H3F7OH+ | 201.0145 | <10 ppt | 0.11 |
| C8H8O6H+ | 201.0394 | <10 ppt | 0.02 |
| C12H8O3H+ | 201.0546 | <10 ppt | 0.02 |
| C9H12O5H+ | 201.0757 | <10 ppt | 0.02 |
| C13H12O2H+ | 201.091 | <10 ppt | 0.02 |
| C10H16O4H+ | 201.1121 | <10 ppt | 0.02 |
| C14H16OH+ | 201.1274 | <10 ppt | 0.02 |
| C11H20O3H+ | 201.1485 | <10 ppt | 0.05 |
| C15H20H+ | 201.1638 | <10 ppt | 0.18 |
| C12H24O2H+ | 201.1849 | <10 ppt | 0.04 |
| C13H28OH+ | 201.2213 | <10 ppt | 0.01 |
| C7H7NO6H+ | 202.0346 | <10 ppt | 0.01 |
| C11H7NO3H+ | 202.0499 | <10 ppt | 0.01 |
| C8H11NO5H+ | 202.071 | <10 ppt | 0.01 |
| C12H11NO2H+ | 202.0863 | <10 ppt | 0.01 |
| C9H15NO4H+ | 202.1074 | <10 ppt | 0.01 |
| C13H15NOH+ | 202.1226 | <10 ppt | 0.01 |
| C10H19NO3H+ | 202.1438 | <10 ppt | 0.01 |
| C14H19NH+ | 202.159 | <10 ppt | 0.03 |
| C11H23NO2H+ | 202.1802 | <10 ppt | 0.03 |
| C12H27NOH+ | 202.2165 | <10 ppt | 0.01 |
| C7H6O7H+ | 203.0186 | <10 ppt | 0.03 |
| C11H6O4H+ | 203.0339 | <10 ppt | 0.03 |
| C8H10O6H+ | 203.055 | <10 ppt | 0.02 |
| C12H10O3H+ | 203.0703 | <10 ppt | 0.02 |
| C16H10H+ | 203.0855 | <10 ppt | 0.02 |
| C9H14O5H+ | 203.0914 | <10 ppt | 0.02 |
| C13H14O2H+ | 203.1067 | <10 ppt | 0.02 |
| C10H18O4H+ | 203.1278 | <10 ppt | 0.11 |
| C14H18OH+ | 203.143 | <10 ppt | 0.28 |
| C11H22O3H+ | 203.1642 | 0.01 | 0.23 |
| C15H22H+ | 203.1794 | 0.06 | 0.70 |
| C12H26O2H+ | 203.2006 | 0.01 | 0.12 |
| C10H5NO4H+ | 204.0291 | <10 ppt | 0.01 |
| C7H9NO6H+ | 204.0503 | <10 ppt | 0.01 |
| C11H9NO3H+ | 204.0655 | <10 ppt | 0.01 |
| C8H13NO5H+ | 204.0867 | <10 ppt | 0.01 |
| C12H13NO2H+ | 204.1019 | <10 ppt | 0.01 |
| C9H17NO4H+ | 204.123 | <10 ppt | 0.01 |
| C13H17NOH+ | 204.1383 | <10 ppt | 0.01 |
| C10H21NO3H+ | 204.1594 | <10 ppt | 0.03 |
| C14H21NH+ | 204.1747 | <10 ppt | 0.08 |
| C11H25NO2H+ | 204.1958 | <10 ppt | 0.04 |
| C10H5O5+ | 205.0132 | <10 ppt | 0.12 |
| C11H8O4H+ | 205.0495 | <10 ppt | 0.02 |
| C8H12O6H+ | 205.0707 | <10 ppt | 0.01 |
| C12H12O3H+ | 205.0859 | <10 ppt | 0.01 |
| C9H16O5H+ | 205.1071 | <10 ppt | 0.01 |
| C13H16O2H+ | 205.1223 | <10 ppt | 0.03 |
| C10H20O4H+ | 205.1434 | <10 ppt | 0.05 |
| C14H20OH+ | 205.1587 | <10 ppt | 0.05 |
| C11H24O3H+ | 205.1798 | <10 ppt | 2.66 |
| C15H24H+ | 205.1951 | 0.03 | 9.46 |
| C10H7NO4H+ | 206.0448 | <10 ppt | 0.01 |
| C11H11NO3H+ | 206.0812 | <10 ppt | 0.01 |
| C12H15NO2H+ | 206.1176 | <10 ppt | 0.01 |
| C13H19NOH+ | 206.154 | <10 ppt | 0.02 |
| C14H23NH+ | 206.1903 | <10 ppt | 0.11 |
| C6H7O8+ | 207.0136 | <10 ppt | 0.04 |
| C10H6O5H+ | 207.0288 | <10 ppt | 0.04 |
| C7H10O7H+ | 207.0499 | <10 ppt | 0.02 |
| C11H10O4H+ | 207.0652 | <10 ppt | 0.02 |
| C8H14O6H+ | 207.0863 | <10 ppt | 0.01 |
| C12H14O3H+ | 207.1016 | <10 ppt | 0.01 |
| C9H18O5H+ | 207.1227 | <10 ppt | 0.01 |
| C13H18O2H+ | 207.138 | <10 ppt | 0.02 |
| C10H22O4H+ | 207.1591 | <10 ppt | 0.52 |
| C14H22OH+ | 207.1744 | 0.01 | 1.53 |
| C15H26H+ | 207.2107 | <10 ppt | 0.06 |
| C10H9NO4H+ | 208.0604 | <10 ppt | 0.01 |
| C14H9NOH+ | 208.0757 | <10 ppt | 0.01 |
| C11H13NO3H+ | 208.0968 | <10 ppt | 0.01 |
| C15H13NH+ | 208.1121 | <10 ppt | 0.01 |
| C12H17NO2H+ | 208.1332 | <10 ppt | 0.01 |
| C13H21NOH+ | 208.1696 | <10 ppt | 0.02 |
| C14H25NH+ | 208.206 | <10 ppt | 0.01 |
| C9H4O6H+ | 209.0081 | <10 ppt | 0.04 |
| C6H8O8H+ | 209.0292 | <10 ppt | 0.17 |
| C10H8O5H+ | 209.0445 | <10 ppt | 0.59 |
| C14H8O2H+ | 209.0597 | <10 ppt | 0.02 |
| C11H12O4H+ | 209.0808 | <10 ppt | 0.02 |
| C15H12OH+ | 209.0961 | <10 ppt | 0.07 |
| C12H16O3H+ | 209.1172 | <10 ppt | 0.31 |
| C16H16H+ | 209.1325 | <10 ppt | 0.30 |
| C13H20O2H+ | 209.1536 | 0.01 | 0.76 |
| C14H24OH+ | 209.19 | <10 ppt | 0.03 |
| C15H28H+ | 209.2264 | <10 ppt | 0.02 |
| C10H11NO4H+ | 210.0761 | <10 ppt | 0.01 |
| C14H11NOH+ | 210.0913 | <10 ppt | 0.01 |
| C11H15NO3H+ | 210.1125 | <10 ppt | 0.01 |
| C15H15NH+ | 210.1277 | <10 ppt | 0.01 |
| C12H19NO2H+ | 210.1489 | <10 ppt | 0.09 |
| C13H23NOH+ | 210.1852 | <10 ppt | 0.01 |
| C14H27NH+ | 210.2216 | <10 ppt | 0.01 |
| C9H6O6H+ | 211.0237 | <10 ppt | 0.03 |
| C13H6O3H+ | 211.039 | <10 ppt | 0.04 |
| C10H10O5H+ | 211.0601 | <10 ppt | 0.02 |
| C14H10O2H+ | 211.0754 | <10 ppt | 0.01 |
| C11H14O4H+ | 211.0965 | <10 ppt | 0.01 |
| C15H14OH+ | 211.1117 | <10 ppt | 0.02 |
| C12H18O3H+ | 211.1329 | <10 ppt | 0.02 |
| C16H18H+ | 211.1481 | <10 ppt | 0.02 |
| C13H22O2H+ | 211.1693 | <10 ppt | 0.15 |
| C14H26OH+ | 211.2056 | 0.04 | 1.55 |
| C15H30H+ | 211.242 | <10 ppt | 0.08 |
| C10H16O5H+ | 217.1071 | 0.02 | 0.43 |
| C9H5O7+ | 225.003 | <10 ppt | 0.07 |
| C6H9O9+ | 225.0241 | 0.01 | 0.17 |
| C13H8NO4+ | 242.0448 | 0.01 | 0.31 |
| C13H10NO5+ | 260.0554 | <10 ppt | 0.11 |

# Supplementary Table 2. Data comparing procedures with and without SES. Maximum detected concentrations during open procedures:

| **name** | **mean [VOC] with SES** | **mean [VOC] without SES** | **Sdev with SES** | **Sdev without SES** | **calculated t score** | **p-value** |
| --- | --- | --- | --- | --- | --- | --- |
| Total VOC | 2142.2 | 1992.4 | 2061.932 | 862.260 | 0.261 | 0.38 |
| HydrogenCyanide | 1.9 | 2.3 | 2.313 | 2.965 | 0.434 | 0.36 |
| Formaldehyde | 48.0 | 29.2 | 91.640 | 27.568 | 0.760 | 0.29 |
| Methanol | 36.5 | 43.2 | 60.819 | 44.758 | 0.347 | 0.37 |
| Acetonitrile | 7.8 | 18.7 | 7.552 | 15.616 | 2.513 | 0.02 |
| Acetaldehyde | 347.5 | 377.5 | 121.030 | 75.265 | 0.822 | 0.28 |
| FormicAcid | 0.5 | 0.5 | 0.678 | 0.220 | 0.270 | 0.38 |
| Ethanol | 294.5 | 198.6 | 524.941 | 229.389 | 0.652 | 0.32 |
| Methanethiol | 0.8 | 1.6 | 1.051 | 1.838 | 1.507 | 0.13 |
| Acrylonitrile | 1.8 | 4.2 | 3.150 | 3.941 | 1.906 | 0.07 |
| Butadiene | 136.7 | 88.7 | 265.173 | 73.655 | 0.678 | 0.31 |
| Propionitrile | 1.7 | 4.0 | 2.700 | 3.494 | 2.124 | 0.05 |
| Butenes | 11.8 | 20.3 | 7.161 | 30.173 | 1.096 | 0.22 |
| Acetone | 137.6 | 189.1 | 95.025 | 121.675 | 1.318 | 0.17 |
| ButeneNitrile | 2.4 | 5.1 | 3.700 | 5.854 | 1.553 | 0.12 |
| Pentadiene | 18.0 | 41.8 | 13.167 | 53.951 | 1.714 | 0.09 |
| Crotonaldehyde | 2.0 | 4.4 | 2.853 | 3.051 | 2.287 | 0.03 |
| Benzene | 2.9 | 6.1 | 5.110 | 4.364 | 1.877 | 0.07 |
| Pyridine | 0.3 | 0.7 | 0.457 | 0.666 | 1.973 | 0.06 |
| MeButanenitrile | 0.5 | 1.4 | 0.758 | 1.556 | 1.955 | 0.06 |
| Methyl2butenal | 0.5 | 1.1 | 0.602 | 0.706 | 2.600 | 0.02 |
| gButyrolactone | 1.7 | 1.9 | 1.004 | 1.006 | 0.631 | 0.32 |
| Isovaleraldehyde | 1.8 | 3.4 | 0.812 | 3.348 | 1.952 | 0.06 |
| Toluene | 1.9 | 2.7 | 2.425 | 1.633 | 1.069 | 0.22 |
| Phenol | 3.8 | 4.9 | 1.541 | 1.831 | 1.903 | 0.07 |
| Furfural | 0.3 | 0.6 | 0.250 | 0.727 | 1.611 | 0.11 |
| Dimethylfuran | 0.3 | 0.7 | 0.317 | 0.489 | 2.957 | 0.01 |
| MeMethacrylate | 0.5 | 0.8 | 0.226 | 0.468 | 2.174 | 0.04 |
| Styrene | 0.5 | 1.0 | 0.589 | 0.719 | 2.064 | 0.05 |
| Indane | 0.2 | 0.3 | 0.130 | 0.215 | 1.779 | 0.08 |
| Propylbenzene | 0.4 | 0.6 | 0.189 | 0.446 | 1.293 | 0.17 |
| Desflurane | 76.2 | 47.7 | 256.803 | 76.605 | 0.412 | 0.36 |
| Sevoflurane | 68.3 | 52.1 | 137.337 | 105.932 | 0.366 | 0.37 |

### Supplementary Table 3. Data comparing procedures with and without SES. Maximum detected concentrations during minimally invasive procedures:

| **name** | **mean [VOC] with SES** | **mean [VOC] without SES** | **Sdev with SES** | **Sdev without SES** | **calculated t score** | **p-value** |
| --- | --- | --- | --- | --- | --- | --- |
| Total VOC | 1450.551 | 1861.299 | 989.319 | 1418.426 | 1.129 | 0.21 |
| HydrogenCyanide | 1.966 | 4.989 | 3.087 | 6.613 | 1.890 | 0.07 |
| Formaldehyde | 34.151 | 128.367 | 86.400 | 388.612 | 1.044 | 0.23 |
| Methanol | 28.195 | 15.839 | 48.113 | 19.724 | 1.356 | 0.16 |
| Acetonitrile | 7.618 | 8.969 | 6.944 | 7.809 | 0.636 | 0.32 |
| Acetaldehyde | 355.238 | 382.524 | 129.770 | 105.507 | 0.846 | 0.28 |
| FormicAcid | 0.808 | 0.250 | 3.076 | 0.077 | 1.103 | 0.22 |
| Ethanol | 174.935 | 125.839 | 286.070 | 110.328 | 0.919 | 0.26 |
| Methanethiol | 0.172 | 0.198 | 0.243 | 0.191 | 0.430 | 0.36 |
| Acrylonitrile | 0.312 | 0.362 | 0.568 | 0.417 | 0.375 | 0.37 |
| Butadiene | 52.396 | 85.594 | 68.815 | 97.874 | 1.320 | 0.17 |
| Propionitrile | 0.366 | 0.472 | 0.637 | 0.428 | 0.738 | 0.30 |
| Butenes | 6.179 | 10.184 | 4.088 | 12.581 | 1.352 | 0.16 |
| Acetone | 135.609 | 135.214 | 86.553 | 60.060 | 0.020 | 0.40 |
| ButeneNitrile | 0.379 | 0.435 | 0.834 | 0.401 | 0.336 | 0.37 |
| Pentadiene | 9.355 | 11.314 | 7.643 | 9.611 | 0.772 | 0.29 |
| Crotonaldehyde | 0.718 | 0.788 | 0.736 | 0.509 | 0.414 | 0.36 |
| Benzene | 1.131 | 1.591 | 1.008 | 1.781 | 1.043 | 0.23 |
| Pyridine | 0.089 | 0.098 | 0.112 | 0.075 | 0.323 | 0.38 |
| MeButanenitrile | 0.164 | 0.174 | 0.260 | 0.136 | 0.184 | 0.39 |
| Methyl2butenal | 0.197 | 0.256 | 0.189 | 0.145 | 1.304 | 0.17 |
| gButyrolactone | 0.892 | 0.931 | 0.409 | 0.362 | 0.360 | 0.37 |
| Isovaleraldehyde | 1.578 | 2.062 | 0.909 | 1.888 | 1.056 | 0.23 |
| Toluene | 0.849 | 0.758 | 0.447 | 0.361 | 0.825 | 0.28 |
| Phenol | 3.098 | 3.145 | 0.620 | 0.793 | 0.226 | 0.39 |
| Furfural | 0.121 | 0.122 | 0.066 | 0.059 | 0.027 | 0.40 |
| Dimethylfuran | 0.133 | 0.157 | 0.142 | 0.078 | 0.813 | 0.28 |
| MeMethacrylate | 0.346 | 0.479 | 0.186 | 0.325 | 1.644 | 0.10 |
| Styrene | 0.262 | 0.290 | 0.200 | 0.237 | 0.444 | 0.36 |
| Indane | 0.125 | 0.138 | 0.038 | 0.065 | 0.761 | 0.30 |
| Propylbenzene | 0.286 | 0.307 | 0.145 | 0.178 | 0.451 | 0.36 |
| Desflurane | 33.145 | 5.024 | 80.470 | 6.231 | 2.113 | 0.04 |
| Sevoflurane | 20.423 | 66.554 | 43.825 | 183.599 | 1.080 | 0.22 |

## Supplementary Table 4. Data comparing procedures with and without SES. Mean detected concentrations during open procedures:

| **name** | **mean [VOC] with SES** | **mean [VOC] without SES** | **Sdev with SES** | **Sdev without SES** | **calculated t score** | **p-value** |
| --- | --- | --- | --- | --- | --- | --- |
| Total VOC | 374.680 | 395.291 | 161.447 | 154.383 | 0.363 | 0.37 |
| HydrogenCyanide | 0.405 | 0.280 | 0.583 | 0.322 | 0.734 | 0.30 |
| Formaldehyde | 8.100 | 5.423 | 10.374 | 4.649 | 0.917 | 0.26 |
| Methanol | 5.549 | 5.898 | 5.586 | 5.930 | 0.169 | 0.39 |
| Acetonitrile | 0.830 | 0.946 | 0.442 | 0.435 | 0.740 | 0.30 |
| Acetaldehyde | 123.282 | 122.107 | 57.005 | 37.972 | 0.067 | 0.39 |
| FormicAcid | 0.162 | 0.186 | 0.077 | 0.074 | 0.880 | 0.27 |
| Ethanol | 24.246 | 34.924 | 18.796 | 50.702 | 0.787 | 0.29 |
| Methanethiol | 0.012 | 0.018 | 0.006 | 0.009 | 1.971 | 0.06 |
| Acrylonitrile | 0.016 | 0.028 | 0.019 | 0.027 | 1.477 | 0.13 |
| Butadiene | 13.204 | 13.000 | 11.477 | 7.119 | 0.059 | 0.39 |
| Propionitrile | 0.029 | 0.052 | 0.017 | 0.022 | 3.203 | 0.00 |
| Butenes | 3.777 | 5.979 | 1.864 | 10.442 | 0.830 | 0.28 |
| Acetone | 38.606 | 40.714 | 13.564 | 14.513 | 0.418 | 0.36 |
| ButeneNitrile | 0.019 | 0.041 | 0.014 | 0.027 | 2.882 | 0.01 |
| Pentadiene | 3.167 | 3.679 | 0.616 | 1.237 | 1.472 | 0.13 |
| Crotonaldehyde | 0.389 | 0.467 | 0.099 | 0.192 | 1.435 | 0.14 |
| Benzene | 0.282 | 0.354 | 0.052 | 0.083 | 2.920 | 0.01 |
| Pyridine | 0.021 | 0.031 | 0.006 | 0.009 | 3.766 | 0.00 |
| MeButanenitrile | 0.014 | 0.024 | 0.004 | 0.009 | 4.030 | 0.00 |
| Methyl2butenal | 0.103 | 0.133 | 0.019 | 0.078 | 1.514 | 0.13 |
| gButyrolactone | 0.625 | 0.708 | 0.138 | 0.290 | 1.028 | 0.23 |
| Isovaleraldehyde | 0.604 | 0.652 | 0.521 | 0.563 | 0.247 | 0.38 |
| Toluene | 0.494 | 0.517 | 0.115 | 0.152 | 0.485 | 0.35 |
| Phenol | 2.682 | 2.945 | 0.449 | 0.657 | 1.305 | 0.17 |
| Furfural | 0.067 | 0.087 | 0.016 | 0.027 | 2.581 | 0.02 |
| Dimethylfuran | 0.055 | 0.074 | 0.014 | 0.042 | 1.736 | 0.09 |
| MeMethacrylate | 0.275 | 0.352 | 0.081 | 0.280 | 1.049 | 0.23 |
| Styrene | 0.115 | 0.137 | 0.032 | 0.100 | 0.833 | 0.28 |
| Indane | 0.084 | 0.090 | 0.021 | 0.024 | 0.797 | 0.29 |
| Propylbenzene | 0.150 | 0.167 | 0.073 | 0.092 | 0.570 | 0.33 |
| Desflurane | 1.309 | 2.121 | 3.080 | 3.277 | 0.711 | 0.31 |
| Sevoflurane | 7.508 | 2.965 | 17.284 | 5.105 | 0.979 | 0.24 |

Supplementary Table 5. Data comparing procedures with and without SES. Mean detected concentrations during minimally invasive procedures:

| **name** | **mean [VOC] with SES** | **mean [VOC] without SES** | **Sdev with SES** | **Sdev without SES** | **calculated t score** | **p-value** |
| --- | --- | --- | --- | --- | --- | --- |
| Total VOC | 386.153 | 471.096 | 137.425 | 240.885 | 1.423 | 0.14 |
| HydrogenCyanide | 0.364 | 0.830 | 0.567 | 1.005 | 1.870 | 0.07 |
| Formaldehyde | 6.626 | 12.231 | 10.638 | 16.489 | 1.345 | 0.16 |
| Methanol | 6.027 | 4.348 | 5.493 | 5.219 | 1.120 | 0.21 |
| Acetonitrile | 1.277 | 1.244 | 0.910 | 0.946 | 0.124 | 0.39 |
| Acetaldehyde | 137.137 | 158.037 | 52.701 | 63.546 | 1.232 | 0.19 |
| FormicAcid | 0.146 | 0.117 | 0.062 | 0.047 | 1.938 | 0.06 |
| Ethanol | 28.274 | 34.764 | 16.888 | 42.758 | 0.637 | 0.32 |
| Methanethiol | 0.006 | 0.007 | 0.003 | 0.005 | 1.166 | 0.20 |
| Acrylonitrile | 0.004 | 0.005 | 0.007 | 0.010 | 0.186 | 0.39 |
| Butadiene | 9.699 | 20.159 | 5.361 | 28.334 | 1.594 | 0.11 |
| Propionitrile | 0.016 | 0.019 | 0.011 | 0.013 | 0.841 | 0.28 |
| Butenes | 2.512 | 4.579 | 1.174 | 6.572 | 1.359 | 0.16 |
| Acetone | 48.549 | 43.396 | 37.922 | 22.129 | 0.641 | 0.32 |
| ButeneNitrile | 0.007 | 0.010 | 0.003 | 0.009 | 1.662 | 0.10 |
| Pentadiene | 2.411 | 2.887 | 0.577 | 0.829 | 2.237 | 0.03 |
| Crotonaldehyde | 0.316 | 0.326 | 0.110 | 0.129 | 0.279 | 0.38 |
| Benzene | 0.266 | 0.266 | 0.068 | 0.107 | 0.001 | 0.40 |
| Pyridine | 0.019 | 0.020 | 0.007 | 0.011 | 0.207 | 0.39 |
| MeButanenitrile | 0.012 | 0.013 | 0.005 | 0.006 | 0.871 | 0.27 |
| Methyl2butenal | 0.081 | 0.097 | 0.020 | 0.046 | 1.470 | 0.13 |
| gButyrolactone | 0.498 | 0.555 | 0.125 | 0.248 | 0.946 | 0.25 |
| Isovaleraldehyde | 0.478 | 0.587 | 0.347 | 0.327 | 1.161 | 0.20 |
| Toluene | 0.472 | 0.465 | 0.141 | 0.201 | 0.129 | 0.39 |
| Phenol | 2.688 | 2.753 | 0.468 | 0.752 | 0.342 | 0.37 |
| Furfural | 0.057 | 0.064 | 0.015 | 0.036 | 0.753 | 0.30 |
| Dimethylfuran | 0.047 | 0.058 | 0.013 | 0.027 | 1.650 | 0.10 |
| MeMethacrylate | 0.230 | 0.306 | 0.091 | 0.158 | 1.947 | 0.06 |
| Styrene | 0.101 | 0.119 | 0.041 | 0.067 | 1.081 | 0.22 |
| Indane | 0.076 | 0.082 | 0.017 | 0.035 | 0.678 | 0.31 |
| Propylbenzene | 0.131 | 0.145 | 0.081 | 0.107 | 0.496 | 0.35 |
| Desflurane | 0.840 | 0.895 | 0.804 | 1.090 | 0.195 | 0.39 |
| Sevoflurane | 2.325 | 6.540 | 5.180 | 16.423 | 1.091 | 0.22 |
